# Tumor-derived extracellular vesicles convey solute transporters to induce bioenergetic dependence shift contributing to treatment resistance

**DOI:** 10.7150/thno.100374

**Published:** 2024-09-30

**Authors:** Jayshree Hirpara, Win Lwin Thuya, Sok-Hwee Cheow, Kanishka Fernando, Jie Qing Eu, Lingzhi Wang, Andrea Li-Ann Wong, Eliza Li Shan Fong, Azhar Bin Ali, Ling-wen Ding, Wu Zhuoran, Yaw-Chyn Lim, Shazib Pervaiz, Boon-Cher Goh

**Affiliations:** 1 Cancer Science Institute of Singapore, National University of Singapore, Singapore.; 2 Department of Pharmacology, National University of Singapore, Singapore.; 3 NUS Center for Cancer Research, Yong Loo Lin School of Medicine, National University of Singapore, Singapore.; 4 Departmental of Biomedical Engineering, National University of Singapore, Singapore.; 5 Department of Haematology-Oncology, National University Hospital, Singapore.; 6 The N. 1 Institute of Health, National University of Singapore.; 7 Department of Physiology, Yong Loo Lin School of Medicine, National University of Singapore, Singapore 117593, Singapore.; 8 Healthy Longevity Translational Research Programme, Yong Loo Lin School of Medicine, National University of Singapore, Singapore.

**Keywords:** Extracellular Vesicles, TKI-resistance, SLC1A5, Tumor Microenvironment, metabolic reprogramming

## Abstract

**Rationale:** Growing evidence points to the tumor microenvironment's role in developing drug resistance. A key element of this microenvironment is inter-cellular communication, which includes the release of membrane-encapsulated vesicles containing various cargo, known as extracellular vesicles (EVs). Understanding how EVs contribute to acquired resistance holds significant clinical implications.

**Methods:** Differential centrifugation-based methods were used to isolate EVs from established cell lines and human plasma. TMT labeling proteomics analysis of EVs revealed an abundance of metabolic transporter proteins. Increased expression of SLC1A5 in EVs of patient-derived plasma and cell lines rendered resistant to tyrosine kinase inhibitors and its relationship with progression-free survival was assessed using Kaplan-Meier survival plot. Gene knockdown and overexpression of *SLC1A5* were used to validate its effect on Tyrosine kinase inhibitor (TKI) resistance. Co-culture assays using inserts was used to evaluate the effect of resistant EVs on normal fibroblasts and epithelial cells. Next, mouse-derived tumor slices (MDTS) were cultured *in vitro* to assess the effect of resistant EVs.

**Results:** We report here that TKI-sensitive cells are rendered resistant upon incubation with EVs derived from TKI-resistant cell lines. Metabolic transporters, in particular SLC1A5 and SLC25A5, are upregulated in EVs derived from TKI-resistant cells and plasma from patients harbouring TKI-resistant tumors and in TKI-resistant cell lines. Furthermore, we also provide evidence for the increased abundance of pSTAT3 and the stemness marker ALDH1A1 upon EV-induced resistance. Notably, resistant EVs trigger phenotypic and functional switching of lung-derived fibroblasts into tumor-associated fibroblasts, significantly increasing their migratory and invasive capacities.

**Conclusions:** Our findings support the role of metabolic transporters within tumor-derived EVs in reshaping the tumor microenvironment to promote therapy resistance, which could have potential diagnostic, prognostic, and therapeutic implications.

## Introduction

Metastatic disease is the main cause of death from cancer. Despite recent strides in precision medicine, which allows the targeting of specific mutations with minimal adverse effects, resistance to targeted therapies, such as tyrosine kinase inhibition (TKI), remains a major therapeutic challenge. Clinical experience dictates that even with exceptional clinical response and minimal residual disease, relapse is an invariable finding 1 arising as a consequence of treatment resistance. Amongst the many mechanisms involved in the acquisition of resistance, metabolic reprogramming has been identified as a cancer hallmark, which is a critical mediator of processes involved in carcinogenesis and its progression and has been associated with the emergence of resistance to treatment 2,3. Though spatially distinct, metastatic lesions in the patient are observed to acquire resistance to treatment in a temporally concordant manner, suggesting that mechanisms of intercellular communication exist. Tumor-derived EVs are released by cancer cells and include exosomes that are membrane-bound vesicles measuring 40-100 nM and carry bioactive molecules including DNA, mRNA, miRNA, protein, and lipids through the circulation as intercellular communicators that can promote tumor proliferation and metastases when taken up by recipient cells 4. In addition to their effect on neighboring and distant cancer cells, they are able to redirect the function of non-malignant cells in the tumor microenvironment towards preparing prometastatic niches, and organotrophism, disrupting vascular integrity, enhancing vascular permeability and mediating immune function blockade. Tumor cells have been reported to produce significantly higher quantities of EVs compared to their normal counterparts 5. Recent research has also demonstrated that exosomes derived from drug-resistant cells can prime sensitive cells to become resistant via transferring micro-RNA, non-coding RNA as well as protein cargo 6-10. These distinctive features of EVs have heightened interest in the study and use of tumor associated EVs for use as biomarkers for identifying refractory disease and disease progression.

On this basis and our recent report demonstrating the role of metabolic reprogramming in conferring resistance to treatment with TKIs, we investigated the involvement of tumor-derived EVs (TDEs) in promoting drug-resistant phenotype in tumors and established a mechanism for this through upregulation of SLC1A5 in EVs of TKI-resistant cancer cell lines as well as in EVs from plasma of patients with TKI-resistant tumors. We show that TDEs from resistant cells (REVs) could induce resistance in TKI-sensitive cells and alter the tumor micro-environment to support tumor resistance. Importantly, gene silencing of *SLC1A5* or its pharmacological inhibition reverted the resistant phenotype, thereby suggesting that SLC1A5 could be a promising diagnostic marker as well as a therapeutic target for TKI resistant cancers.

## Results

### Upregulation of metabolic pathway-related proteins in EVs derived from plasma of patients with TKI-resistant tumors and cell lines

**Figure 1:**
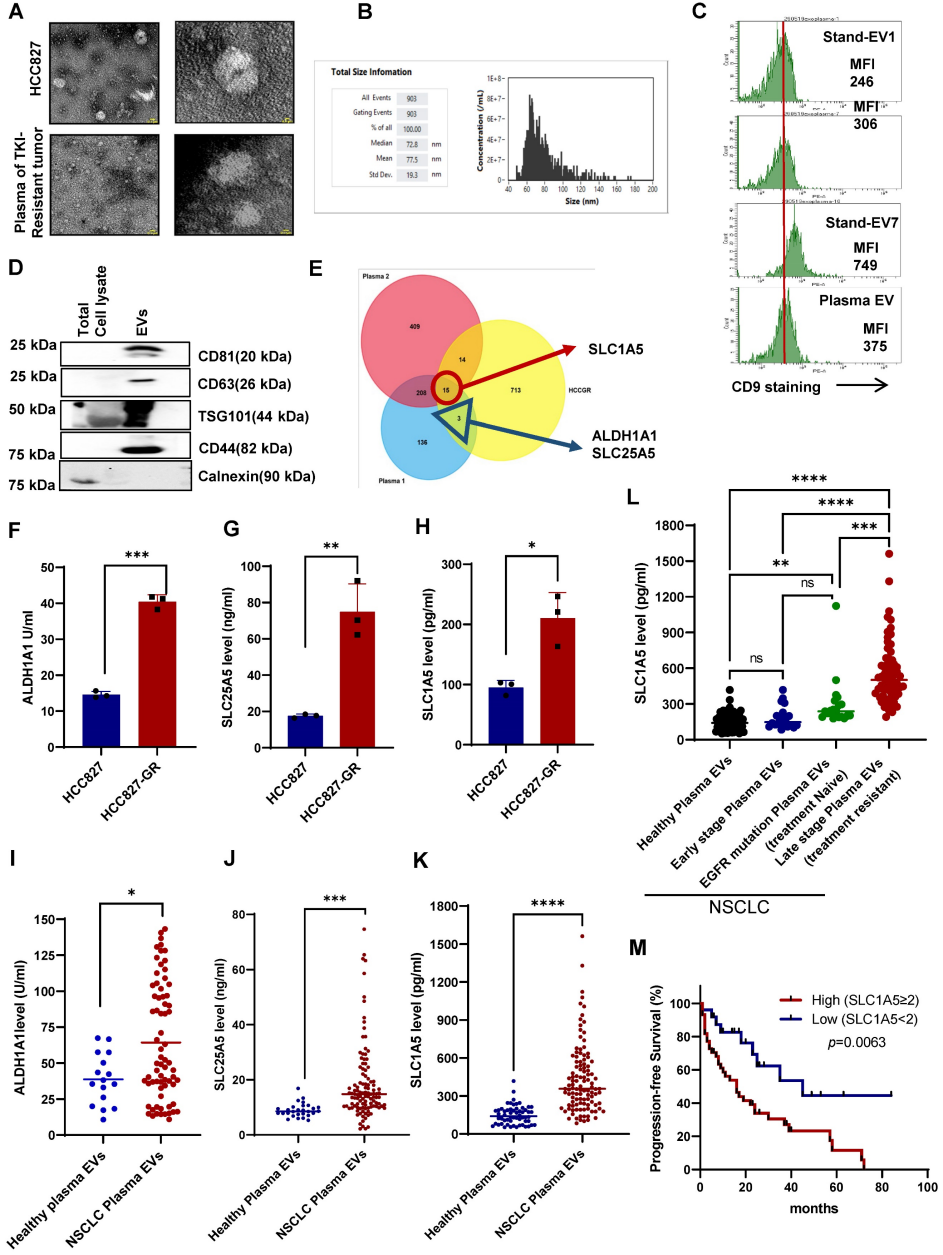
** Upregulation of metabolic pathway-related proteins in EVs derived from TKI-resistant cell lines and patient plasma. (A)** Characterization of EVs isolated from the supernatant of HCC827 and plasma of a patient with TKI-resistant tumor by EM. Scale bar: 0.1 µM. **(B)** Size distribution of EVs isolated from cell culture supernatant analyzed by NanoFC. **(C)** Flow cytometry analysis of CD9 surface expression (MFI: Mean fluorescence intensity) on isolated EVs from plasma of a patient with TKI-resistant tumor. **(D)** Western blot showing expression of CD61, CD81, CD44, TSG101, and calnexin in whole cell lysates and isolated EVs. **(E)** Venn diagram showing the number of upregulated proteins in three different proteomics datasets from EVs of HCC827-GR/HCC827, plasma 1 (TKI-resistant NSCLC/healthy donor), and plasma 2 (pooled plasma of 5 different tumor/healthy donors). **(F, G, and H)** Protein expression of SLC1A5, SLC25A5, and ALDH1A1 in EVs isolated from supernatant of HCC827 and HCC827-GR cells using ELISA as described in Materials and Methods. **(I, J, and K)** Protein expression of SLC1A5, SLC25A5, and ALDH1A1 in the EVs isolated from the plasma of healthy donors (n = 16 for ALDH1A1 and n = 28 for SLC25A5 and n = 58 for SLC1A5) and plasma of TKI-resistant NSCLC patients (n = 111) by ELISA as described in Materials and Methods. **(L)** Protein expression of SLC1A5 in the EVs isolated from the plasma of healthy donors (n = 58), plasma of early-stage NSCLC (n = 20), plasma of EGFT mutant and treatment naïve NSCLC (n = 21), and late-stage and treatment-resistant NSCLC (N = 62) by ELISA as described in Materials and Methods. **(M)** Progression-free survival curve was generated using NSCLC patient's survival details with SLC1A5 level in fold difference (high SLC1A5 level ≥ 2, n = 44 and low SLC1A5 ≤ 2, n = 26 Log-rank Hazard Ratio: 2.632, 95% CI, 1.441-4.807, *p* = 0.0063). Unpaired T-test and two-way Anova were used in GraphPad Prism, version 9 for statistical significance (*p < 0.05, **p < 0.01, ***p < 0.001, ****p < 0.0001, ns: not significance).

To identify components of cell-derived EVs secreted by human cancer NSCLC cells or in the plasma of patients with TKI resistance tumors, we isolated EVs from the supernatant of cancer cell lines (Non-small lung cancer cell line: HCC827, HCC827-GR, Malignant melanoma: A375, A375-VR) as well as from plasma of healthy donors or patients with NSCLC. EVs were characterized structurally by transmission electron microscopy (TEM) and flow cytometry using Nano FC and nanoparticle tracking analyzer (NTA). TEM data showed cup-shaped particles approximately around 50-100 nM in diameter (Figure 1A), while NTA revealed particle sizes between 50-150 nM (Figure S1A), which was corroborated by cytometry using Nano FC (Figure 1B). In addition, EVs were further validated by the surface expression of CD9, a bonafide EV marker (Figure 1C). Lastly, EV-specific markers such as CD63, CD81, CD44, and TSG101 were present in the isolated EVs (Figure 1D).

Next, to identify and quantitate differentially expressed proteins in the plasma EVs between metastatic treatment-resistant and healthy, we capitalise on TMT (Tandom mass tag) mass spectrometry which can distinguish peptidomic landscape between contrasting disease states. We therefore pooled plasma from patients with 5 different cancers at the point of treatment resistance and compared it with plasma from healthy volunteer. A comparative proteomics analysis was performed on EVs derived from HCC827-GR and HCC827 cells, circulating EVs from the plasma of TKI-resistant NSCLC patients, and EVs from the pooled plasma of 5 different types of metastatic tumors (2 NSCLC, 1 Breast Ca, 1 Ovarian Ca, and 1 Colon Ca) compared to healthy donors. By mass spectrometry using 10 plex TMT labeling, a total of 5934 proteins were quantified and identified after filtering, and representative MS spectra data for EVs proteins are summarized in Table S1. A total of 750 proteins were upregulated (fold change > 1.2) while 539 proteins were downregulated (fold change < 0.8) in the HCC827GR-EVs compared to HCC827-EVs (Figure S1B). For NSCLC plasma EVs a total of 647 proteins (Figure S1C) and for pooled plasma EVs 790 proteins showed significant changes compared to EVs from healthy donors (Figure S1D).

Commonly upregulated proteins in all three different datasets (HCCGR: Resistant EVs from the resistant cell lines, Plasma 1: EVs from the single TKI resistant NSCLC, and Plasma 2: EVs from pooled plasma of 5 different types of patients with metastatic tumors) are shown in the Venn diagram (Figure 1E, Table S2). After employing a stringent cutoff in the metabolic proteins, 3 proteins were shortlisted, namely, solute carrier family protein 1 SLC1A5 (ASCT2), a neutral amino acid transporter and well-known glutamine transporter, ALDH1A1, Aldehyde dehydrogenase 1, stem cell marker and SLC25A5, ATP/ADP transporter. While SLC1A5 was upregulated in all three datasets, ALDH1A1 and SLC25A5 were upregulated in EVs from NSCLC plasma and cell lines (Figure 1E). Next, the expression of the 3 shortlisted proteins in the EVs from cell lines was verified using ELISA (Figure 1F-H). Importantly, the expression of SLC1A5, SLC25A5, and ALDH1A1 was validated using circulating EVs from the plasma of an orthogonal sample set of 40 different TKI NSCLC patients compared with 20 healthy donors. All three proteins were significantly upregulated in the TKI-resistant patient plasma EVs compared to healthy donors' plasma EVs (Figure S1E-F). We further escalated our study with an independent dataset of 111 TKI-resistant NSCLC and 28 healthy donors and investigated the expression of proteins in plasma EVs by ELISA. The resulting data further validated the higher level of SLC25A5, and ALDH1A1 in the plasma EVs of patients with TKI-resistant NSCLC (Figure 1I-J). SLC1A5 was upregulated on all three data sets so we further validated the increase in SLC1A5 in another population and increased the sample size and we found significantly higher levels of SLC1A5 in the plasma EVs of patients with NSCLC (Figure 1K). Compared with healthy individuals, early-stage (I and II) NSCLC patients had similar concentrations of SLC1A5 while late-stage treatment naïve patients with EGFR mutation-positive NSCLC had slightly elevated SLC1A5. Conversely, plasma EVs of patients with acquired resistance to EGFR TKI have a significantly higher SLC1A5 compared with the other 3 cohorts (Figure 1L). Of consequence, the progression-free survival (PFS) of patients with higher SLC1A5 in plasma EVs is shorter than those with lower SLC1A5, suggesting that SLC1A5 is positively associated with treatment resistance (Hazard Ratio: 2.632, 95% CI, 1.441 - 4.807, *p* = 0.0063) (Figure 1M). Taken together, these results have unraveled potential biomarkers that are upregulated in TKI-resistant tumors and secreted in plasma EVs of patients derived from TKI-resistant tumors.

### EVs from TKI-resistant tumors induce resistance in TKI-sensitive cells

**Figure 2:**
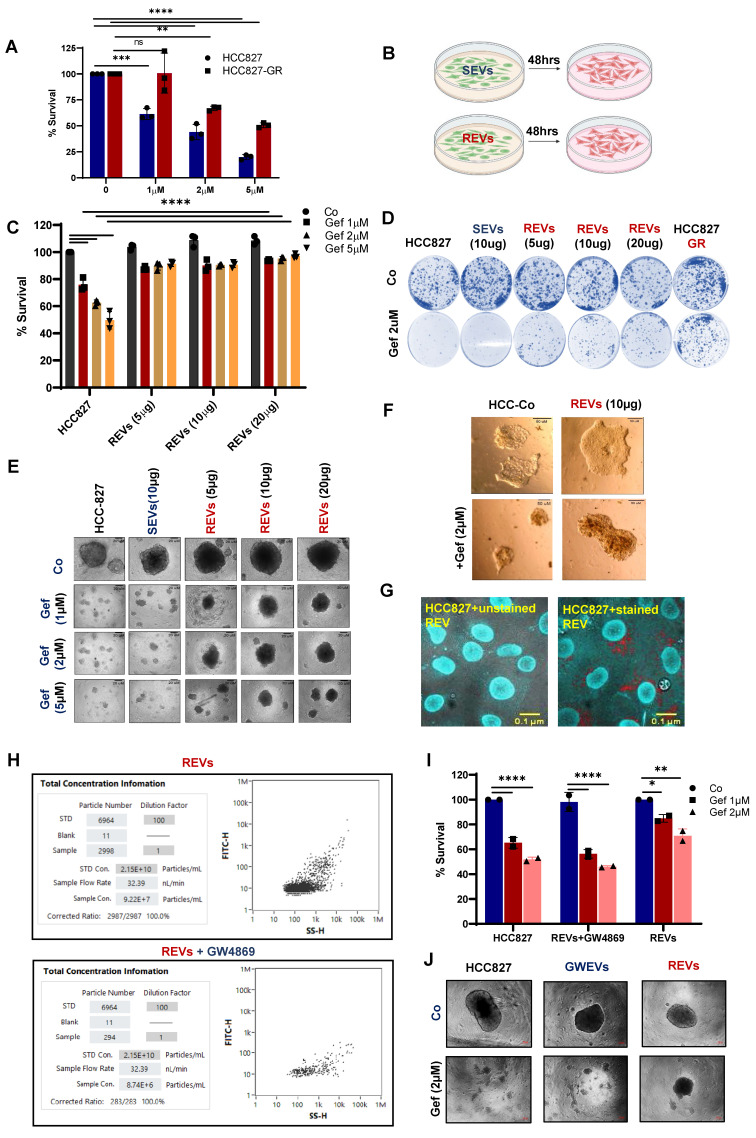
** EVs from TKI-resistant tumors induce resistance in TKI-sensitive cells (A)** Cell viability of HCC827 and gefitinib-resistant HCC827-GR cells following 24 hours treatment with increasing concentrations of gefitinib and measured by crystal violet assay. **(B)** Schematic diagram made using Biorender software showing the experimental plan for exposure of TKI-sensitive cells to SEVs and REVs for 48 hours for use in further analyses. **(C)** HCC827 cells were exposed to SEVs or increasing concentrations of REVs for 48 hours followed by gefitinib treatment for 24 hours and cell viability was measured by crystal violet staining. **(D)** Effects on tumor long-term colony formation, **(E)** Spheroid formation, and **(F)** spheroid formation in matrigel were also assessed. 3000 cells (from the setup shown above) were re-seeded on 6-well plates or low attachment spheroid plates or small spheroids from the hanging drop method were seeded in matrigel and left for 7-10 days before staining with crystal violet or viewing under the microscope under 10X magnification, respectively. (Scale bar: 20 µM and 50 µM respectively). **(G)** Isolated EVs were stained with Acoerela for 1 h and the stained EVs were incubated with HCC827 cells for 6 hours, and visualized by confocal microscopy (Scale bar: 0.1 µM). **(H)** EVs concentration was checked using NanoFC after isolating from HCC827-GR cells treated with or without 10 µM GW4869 (GWEVs) for 48 hours. **(I)** HCC827 cells were exposed to GWEVs and REVs for 48 hours followed by treatment with gefitinib for 24 hours and cell viability was assessed by crystal violet staining. **(J)** Spheroid formation was assessed by re-seeding 3000 cells on low attachment spheroid plates and left for 7-10 days before viewing under the microscope using 10X magnification (Scale bar: 100 µM). Data are representative of at least 2-3 independent experiments and shown as mean ± SD of biological triplicates. Two-way ANOVA was employed for statistical significance (*p < 0.05, **p < 0.01, ***p < 0.001, ****p < 0.0001, ns: not significance).

Our finding of higher concentration of membrane solute carrier proteins in drug-resistant exosomes led to the hypothesis that these EVs may be mediators of treatment resistance between cells. Of note, metabolic reprogramming of sensitive cells is a resistance mechanism that has been reported 11,12. Our recent publication also provides testimony that the acquisition of TKI resistance results in metabolic reprogramming 3. To do so, we employed HCC827 cells that are sensitive to gefitinib and their resistant counterpart, HCC827-GR (Figure 2A). First, we isolated EVs from the supernatant of cultured TKI-sensitive (HCC827: gefitinib sensitive, and A375: vemurafenib sensitive) and their derived corresponding TKI-resistant (HCC827-GR: gefitinib resistant, and A375-VR: vemurafenib resistant) cells. EVs were isolated from the supernatants of TKI-resistant cells (REVs) and TKI-sensitive cells (SEVs) were exposed to TKI-sensitive cells (Figure S1A, 2B). HCC827 and A375 cells were first exposed to different concentrations of REVs derived from HCC827-GR or vemurafenib-resistant A375 cells (A375-VR) for 48 hours followed by treatment with different concentrations of gefitinib and vemurafenib for 24 hours. Results show remarkable induction of drug resistance in the TKI-sensitive cells after exposure to REVs in a concentration-dependent manner compared to SEVs (Figure 2C, S2B). From tumor spheroid formation and colony formation similar results were obtained (Figure 2D-E, S2C). These findings were further corroborated in spheroid Matrigel assays where HCC827 cells were rendered resistant to gefitinib after exposure to REVs. (Figure 2F).

To examine if the isolated EVs were internalized by the host cells or in a paracrine manner by the surrounding cells, isolated EVs were labeled with fluorescent tags, PKH-26 or Acorella, and host cells were co-cultured with the fluorescent-labeled EVs for 6 hours. Confocal microscopy confirmed that isolated EVs were internalized or taken up by the recipient cells (Figure 2G, S2D).

Stimulated by these results, we questioned whether inhibiting the release of EVs prevented the effect of REV on sensitive cells. To do so, we employed GW4869, a sphingomyelinase inhibitor that has been shown to inhibit exosome release from multivesicular bodies 13. HCC827-GR cells were treated with 10 µM GW4869 for 48 hours and EVs were isolated from the cell culture supernatant. First, results show a significant decrease in the concentration of EVs upon exposure to GW4869 (Figure 2H). Importantly, EVs from GW4869-treated HCC827-GR cells restored gefitinib sensitivity (Figure 2I-J), thereby indicating that TKI resistance in sensitive cells is primed by EVs from the resistant cells.

### Resistant cell-derived EVs upregulate metabolic transporters

**Figure 3:**
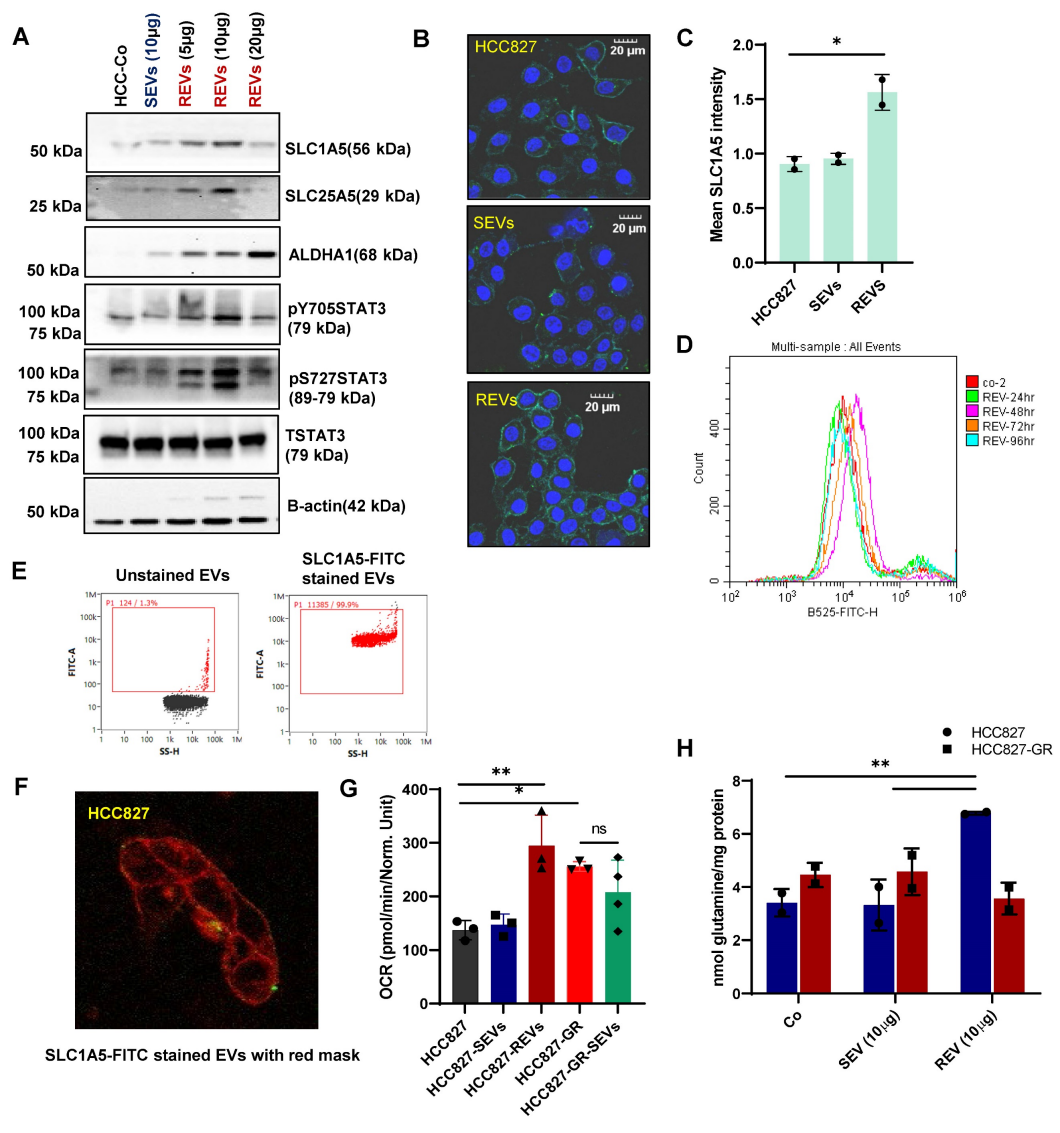
** REVs upregulate metabolic transporters. (A)** Western blot showing increased SLC1A5, SLC25A5, ALDH1A1, STAT3pY705, and STAT3pS727 levels in total lysates of HCC827 cells exposed for 48 hours to SEVs or increasing concentrations of REVs. Total STAT3 and β-actin were used as loading controls. **(B)** Image viewed at 40X magnification by confocal microscopy showing increased surface expression of SLC1A5 in HCC827 cells after 48 hours exposure to REVs (Scale bar: 20 µM). **(C)** Samples from B (individual wells) were scanned using Tissue Fax and mean SLC1A5 intensity was derived and plotted using ImageJ and GraphPad prism, respectively. **(D)** Flow cytometry data showing surface expression of SLC1A5-FITC on HCC827 cells following incubation with 10 µg of REVs for the indicated duration. For flow analysis, at least 10,000 cells were analyzed by flow cytometry as described in Materials and Methods. **(E)** REVs were stained with SLC1A5-FITC and staining was analysed using NanoFC. **(F)** SLC1A5-FITC stained REVs were added to HCC827 cells and live imaging was done using a confocal microscope from 4 to 36 hours for every 20 minutes. A cell mask (red) was used to stain the cell membrane. **(G)** Increase in OCR in HCC827 cells upon exposure to REVs, measured using seahorse and plotted using GraphPad Prism software. **(H)** Increased intracellular glutamine levels in HCC827 cells after 48 hours exposure to REVs. Glutamine was measured as described in materials and methods and plotted using GraphPad prism software. Data are representative of at least 2-3 independent experiments and shown as mean ± SD of biological triplicates. Two-way ANOVA was employed for statistical significance (*p < 0.05, **p < 0.01).

Since EVs from resistant cancers and plasma of TKI-resistant patients were found to carry an abundance of solute carrier membrane proteins, we hypothesized that these SLCs could be taken up by sensitive cells resulting in the conferment of a drug-resistant phenotype. To examine this, we incubated sensitive HCC827 cells at different concentrations of REVs for 48 hours and then measured the expression of proteins including solute carrier membrane proteins. We observed concentration-dependent increases in the expression of SLC1A5, SLC25A5, and ALDH1A1 upon exposure to REVs (Figure 3A). Given recent findings that STAT3 is associated with TKI-resistance 3,14 and upregulates solute carrier proteins and the STAT3-MYC axis has been reported to regulate SLC1A5 to promote stem cell survival 15, we assessed STAT3 activation after REV-mediated induction of resistance. Interestingly, while STAT3 phosphorylation remains unchanged for SEV, upregulation at both Y705 and S727 was observed in REVs (5, 10, 20µg). To further corroborate the effect of resistant EVs on the cells, we expanded our work to a different cell line model i.e., vemurafenib-resistant A375 (A375-VR), and obtained similar results (Figure S3A). To evaluate the effect on the cytokine receptor - STAT3 axis, we measured IL-6 expression and found that REVs exposure was associated with increased IL-6 in a concentration-dependent manner. Conversely, there was no effect on IL-6 with SEVs exposure (Figure S3B).

As SLC1A5 was upregulated in EVs from all TKI-resistant models and SLC1A5 is a membrane transporter, we proceeded to verify its surface expression. Results clearly show that exposure of HCC827 with REVs resulted in a significant increase in membrane expression of SLC1A5 by confocal microscopy and flow cytometry analyses (Figure 3B-D). It should also be pointed out that REVs did not induce a significant effect on *SLC1A5* transcription even with prolonged time observation (Figure S3C), thus indicating that the effect on SLC1A5 was at the post-transcriptional level. To understand this association of REV exposure with enhanced expression of SLC1A5 in HCC827 cells, we stained the REVs with SLC1A5-FITC (Figure 3E) and performed live imaging every 30 minutes from 2-36 hours of incubation with HCC827 cells. SLC1A5 could be seen to be located on the cell membrane of incubated cells (Figure 3F, S3D).

To demonstrate the functional effects of this, we assessed mitochondrial activity and showed that REVs induced an increase in OCR (Oxygen consumption rate) (Figure 3H) as well as mitochondrial superoxide (O_2_^.-^) production with minimal effect on mitochondrial transmembrane potential (Figure S3E-F). Taken together, these data indicate a critical involvement of SLC1A5 in the acquired resistance upon exposure of TKI sensitive cells to REVs.

### REV-induced TKI resistance involves crosstalk between the glutatmine pathway and STAT3 activation

It was previously established that SLC1A5 plays a very important role as a glutamine transporter in cancer cell metabolism 16,17. Our proteomics as well as *in vitro* data also showed increased expression of SLC1A5 in the EVs derived from the resistant cells. We hypothesized that SLC1A5 is transferred from REVs to cell membrane of parental drug sensitive cells could promulgate metabolic changes by facilitating an increased influx of glutamine. Therefore, we measured the levels of intracellular glutamine in the cells after the incorporation of REVs and SEVs.

**Figure 4:**
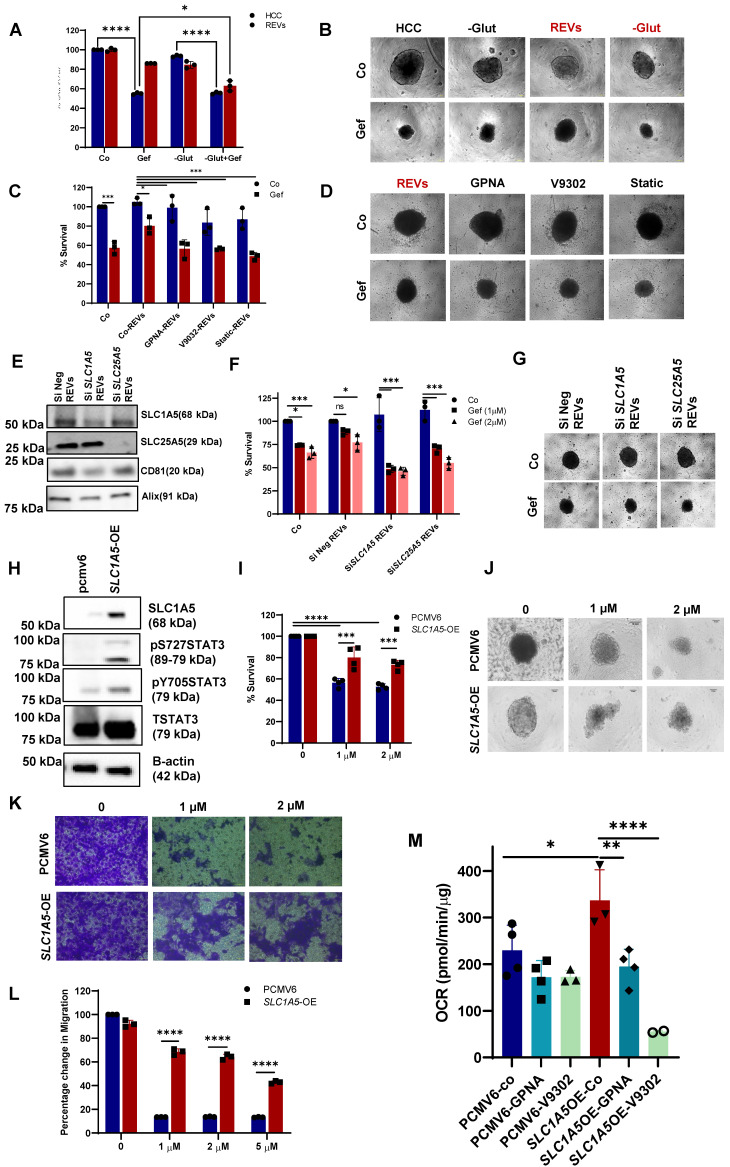
** REV-induced TKI resistance involves crosstalk between the glutamine pathway and STAT3 activation. (A)** HCC827 cells were pre-exposed with REVs for 48 hours before 24 hours of treatment with gefitinib (2µM) in the presence or absence of glutamine. Cell viability was measured using CCK-8 assay. **(B)** Effect on spheroid formation was assessed in the samples from A; 3000 cells were re-seeded on low attachment spheroid plates and left for 7-10 days before viewing under the microscope using 10X magnification (Scale bar: 100 µM). **(C)** REVs pre-exposed HCC827 cells were pre-treated with glutamine transport inhibitors, GPNA (10 µM), V9302(10 µM), and STAT3 inhibitor, STATIC (1 µM), for 1 h before exposure to gefitinib (2 µM) for 24 hours. Cell viability was measured using CCK-8 assay. **(D)** Effect on spheroid formation was assessed in the samples from C; 3000 cells were re-seeded on low attachment spheroid plates and left for 7-10 days before viewing under the microscope using 10X magnification (Scale bar: 100 µM). **(E)** Western blot showing expression of SLC1A5, and SLC25A5 in lysates of EVs isolated from HCC827-GR cells transiently transfected (48 hours) with Si*SLC1A5* and Si*SLC25A5*. CD81 and Alix were used as EV markers. **(F)** HCC827 cells were pre-exposed with REVs isolated from the Si*SLC1A5* and Si*SLC25A5* transfected cells for 48 hours before treatment with gefitinib for 24 hours. Cell viability was measured by crystal violet staining. **(G)** Effect on spheroid formation was assessed in the samples from F; 3000 cells were re-seeded on low attachment spheroid plates and left for 7-10 days before viewing under the microscope using 10X magnification (Scale bar: 100 µM). **(H)** HCC827 cells were stably transfected with pcmv6 vector or SLC1A5-GFP plasmid. Western blot showing over-expression of SLC1A5 and increased STAT3pY705 and STAT3pS727 levels. Total STAT3 and β-actin were used as loading controls. **(I)** Cell viability of HCC827 cells transfected with pcmv6 and *SLC1A5*-OE following treatment with gefitinib for 24 hours. Cell viability was measured using CCK-8 assay. **(J)** Effect on spheroid formation was assessed in the samples from I; 3000 cells were re-seeded on low attachment spheroid plates and left for 7-10 days before viewing under the microscope using 10X magnification (Scale bar: 100 µM). **(K)** Pcmv6 transfected or SLC1A5 overexpressing cells were treated with gefitinib for 24 hours and 75,000 cells were re-seeded into ThinCert® cell culture inserts for 48 hours, stained with crystal violet, and viewed under a microscope (Scale bar: 100 µm) and **(L)** quantified by dissolving with 33% (v:v) acetic acid and measuring absorbance at 590 nm, as described in Materials and Methods. Migration rates are plotted in percentages with respect to control cells. **(M)** Increased OCR in HCC827-*SLC1A5*-OE cells was inhibited upon exposure to glutamine transport inhibitors, GPNA and V9302, measured using seahorse and plotted using GraphPad Prism software. Data are representative of at least 3 independent experiments and shown as mean ± SD of biological triplicates. Two-way ANOVA was employed for statistical significance (*p<0.05, **p < 0.01, ***p < 0.0001, ****p < 0.0001, ns: not significant).

Indeed, elevated levels of glutamine were observed in REV-treated cells, thereby suggesting active SLC1A5 (Figure 3H, S3G). To gain further insight into the involvement of glutamine in REV-induced TKI resistance, we treated HCC827 cells with SEVs or REVs and studied the effect of removing glutamine from the culture medium on sensitivity to gefitinib. The removal of glutamine rescued the effect of REVs on gefitinib sensitivity as shown by effects on cell viability and spheroid formation (Figure 4A-B). In addition, V9302 and GPNA, glutamine transport inhibitors, significantly sensitized REV-treated HCC827 or A375 cells to the respective TKIs (Figure 4C-D and Figure S4A-B). To corroborate these findings, we knocked down the expression of *SLC1A5* and *SLC25A5* by siRNA transfection in HCC827-GR cells (Figure S4C) and isolated EVs from supernatants of si*SLC1A5* and si*SLC25A5* transfected HCC827-GR cells (Figure 4E). EVs derived from cells following gene knockdown of *SLC1A5* (*SLC1A5*-EVs) or *SLC25A5* (*SLC25A5*-EVs) negated the effect of REVs on gefitinib sensitivity (Figure 4F-G), thus highlighting the importance of the glutamine transporter in REV-induced acquired resistance to TKI.

In a separate set of experiments, we also investigated the crosstalk between glutamine uptake and STAT3 activation, observed upon exposure to REVs. Similar to pharmacological inhibitors of glutamine uptake, STAT3 inhibition (stattic) also blocked the effect of REVs on gefitinib sensitivity, as shown by cell viability and spheroid formation (Figure 4C-D). Interestingly, whereas inhibiting glutamine uptake significantly blocked STAT3 phosphorylation, inhibition of STAT3 activation also prevented the effect of REVs on SLC1A5 and SLC25A5 (Figure S4D). The link between STAT3 phosphorylation and SLC1A5 was further corroborated in HCC827-*SLC1A5*-GFP overexpressing cells (*SLC1A5*-OE); STAT3 phosphorylation was significantly upregulated in *SLC1A5*-OE cells (Figure 4H), thus validating earlier published results 18. Also, *SLC1A5*-OE cells were resistant to gefitinib, compared to the parental HCC827 cell line (Figure 4I-J), as well as negated the effect of gefitinib on cell migration (Figure 4K-L). Moreover, *SLC1A5*-OE cells exhibited significantly increased OCR, which could be blocked by glutamine inhibitors, thus corroborating the important role of glutamine in mitochondrial OXPHOS (Figure 4M). Together, these data point to the critical involvement of glutamine transport in REV-induced resistance to TKI as well as support interplay between two critical pathways involved in acquired resistance to TKIs.

**Figure 5:**
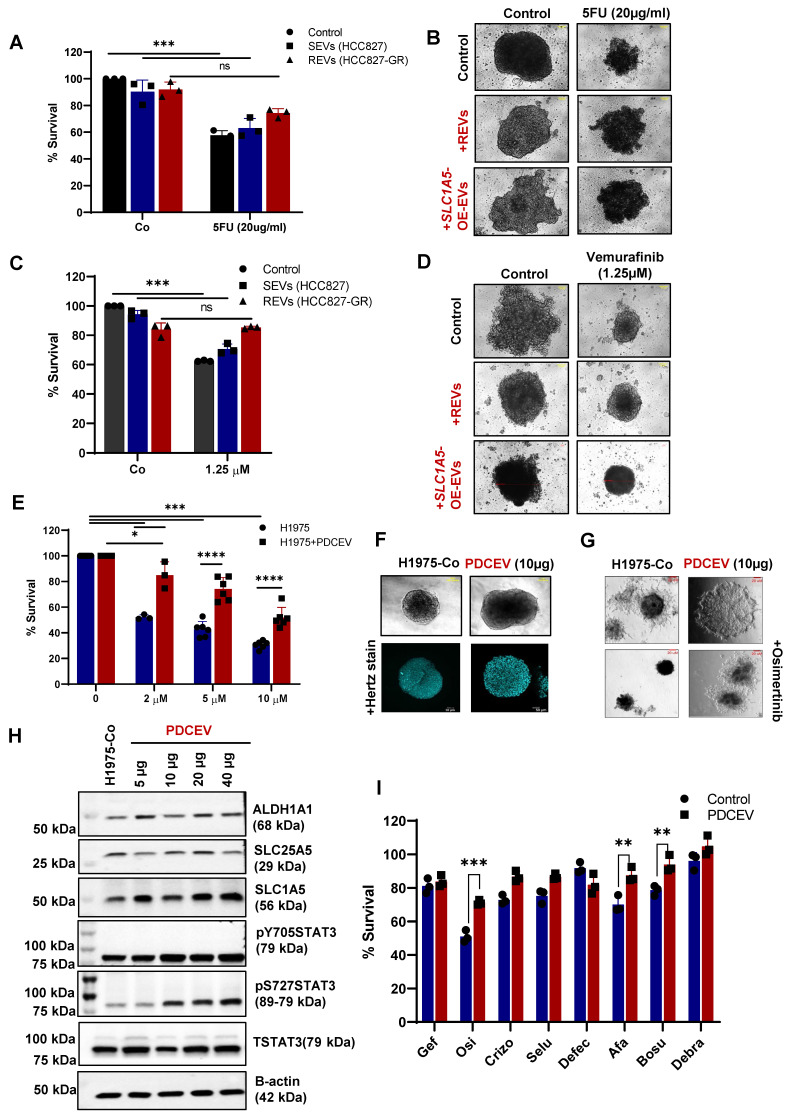
** REVs induce resistance in different tumor models. (A)** HCT116 cells were pre-exposed with SEVs, REVs, and *SLC1A5*-OE-EVs for 48 hours before treatment with 5 FU for 24 hours. Cell viability was measured using CCK-8 assay. **(B)** Effect on spheroid formation was assessed in the samples from A; 3000 cells were re-seeded on low attachment spheroid plates and left for 7-10 days before viewing under the microscope using 10X magnification (Scale bar:10 µm). **(C)** A375 cells were pre-exposed to SEVs, REVs, and *SLC1A5*-OE-EVs for 48 hours before treatment with Vemurafinib for 24 hours. Cell viability was measured using CCK-8 assay. **(D)** Effect on spheroid formation was assessed in the samples from C; 3000 cells were re-seeded on low attachment spheroid plates and left for 7-10 days before viewing under the microscope using 10X magnification (Scale bar:10 µm). **(E)** H1975 cells were pre-exposed to 10 µg EVs isolated from patient-derived cell lines (PDCEVs) for 48 hours before treatment with Osimertinib for 24 hours. Cell viability was measured using CCK-8 assay. **(F, G)** Spheroid formation in matrigel was assessed in the samples from E using the hanging drop technique (seeded in Matrigel) and left for 7-10 days before viewing under the microscope using 10X magnification or analyzed by confocal microscopy, respectively (Scale bar: 50 µM or 20 µM, respectively). **(H)** Western blot showing increased SLC1A5, SLC25A5, ALDH1A1, STAT3pY705, and STAT3pS727 levels in total lysates of H1975 cells exposed for 48 hours with increasing concentration of PDCEVs. Total STAT3 and B-actin were used as loading controls. **(I)** H1975 cells were pre-exposed with PDCEVs for 48 hours before treatment with 2 µM Gefitinib, Osimertinib, Crizotinib, Selumetinib, Afatinib, Bosutinib, and Debrafenib for 24 hours. Cell viability was measured using CCK-8 assay. Data are representative of at least 3 independent experiments and shown as mean ± SD of biological triplicates. Two-way ANOVA was employed for statistical significance (**p < 0.01, ***p < 0.001, ns: not significant).

EVs-mediated drug resistance has been reported for many therapies like chemotherapy, radiotherapy, immunotherapy, etc. Therefore, we also checked if REVs or EVs from *SLC1A5*-OE (*SLC1A5*-OE-EVs) (Figure S4E-F) cells induced drug resistance in different tumor cell types. A significant inhibitory effect of REVs and *SLC1A5*-OE-EVs was observed on the sensitivity of HCT116 (Colon cancer cell line) cells to 5-FU (Figure 5A-B) and on vemurafenib sensitivity of A375 cells (Figure 5C-D). These findings suggest that EVs from TKI-resistant cells retain the ability to induce acquired resistance across cell types in a generalized way.

### EVs from TKI resistant patient enhance resistance and upregulate metabolic transporters

To further confirm our findings on clinically relevant tumors, we used EVs from the supernatant of a patient-derived cell line (PDCEVs) carrying T790 mutation and with clinical evidence of resistance to osimertinib. NTA (Nanoparticle tracking analysis) of EVs isolated by ultracentrifugation revealed a mean particle size of 200 nm (Figure S5A).

H1975 cells carrying the T790 mutation were incubated with 10 µg of PDCEVs for 48 hours, re-plated, and treated with different concentrations of osimertinib. Data show that PDCEVs significantly blocked the effect of osimertinib on H1975 cells' viability (Figure 5E). These effects were confirmed using tumor spheroid assays on matrigel (Figure S5B and Figure 5F-G); PDCEVs inhibited the effect of osimertinib on tumor spheroid formation. Furthermore, a dose-dependent effect of PDCEVs (5-40 μg for 48 hours) on SLC1A5, ALDH1A1, SLC25A5, and pSTAT3 was observed in H1975 cells (Figure 5H), which supports our data linking REVs to acquired TKI resistance in HCC827 cells.

Next, we checked whether resistance induced by REVs is exclusive to the specific TKIs or applies universally to all TKIs. To integrate that, we treated PDCEVs exposed H1975 cells and REVs exposed HCC827 cells to 6 different inhibitors (crizotinib, defactinib, selumetinib, afatinib, bosutinib and debrafenib). Interestingly, cell viability data shows significant inhibition with 2^nd^ and 3^rd^ generation TKI in H1975 cells (Figure 5I) whereas, HCC827 cells were resistant to 1st-generation TKI only (Figure S5C). These results suggest that REVs-induced resistance is exclusive to the respective TKI and does not seem to be a universal phenomenon. The mechanism of this selectivity remains to be further investigated.

As our proteomics analysis revealed upregulation of metabolic proteins in REVs, we performed RNA Seq analysis on RNA extracted from H1975 cells with or without exposure to PDCEVs. The volcano graph shows the upregulation of 393 transcripts and downregulation of 270 transcripts upon exposure to PDCEVs, compared to RNA from untreated H1975 cells (Figure S5D). According to GO (Gene Ontology) analysis, the most significantly upregulated transcripts in response to PDCEVs include cellular nitrogen compound metabolic processes, biosynthetic processes, DNA binding activity etc. (Figure S5E), while the KEGG (Kyoto Encyclopaedia of Genes and Genomes) database also revealed metabolic pathways as the highest enriched upon exposure to PDCEVs (Figure S5F). These data confirm the upregulation of metabolic pathways upon exposure to REVs and validate our proteomic findings.

### Effect of REVs on cellular components of the tumor microenvironment

**Figure 6:**
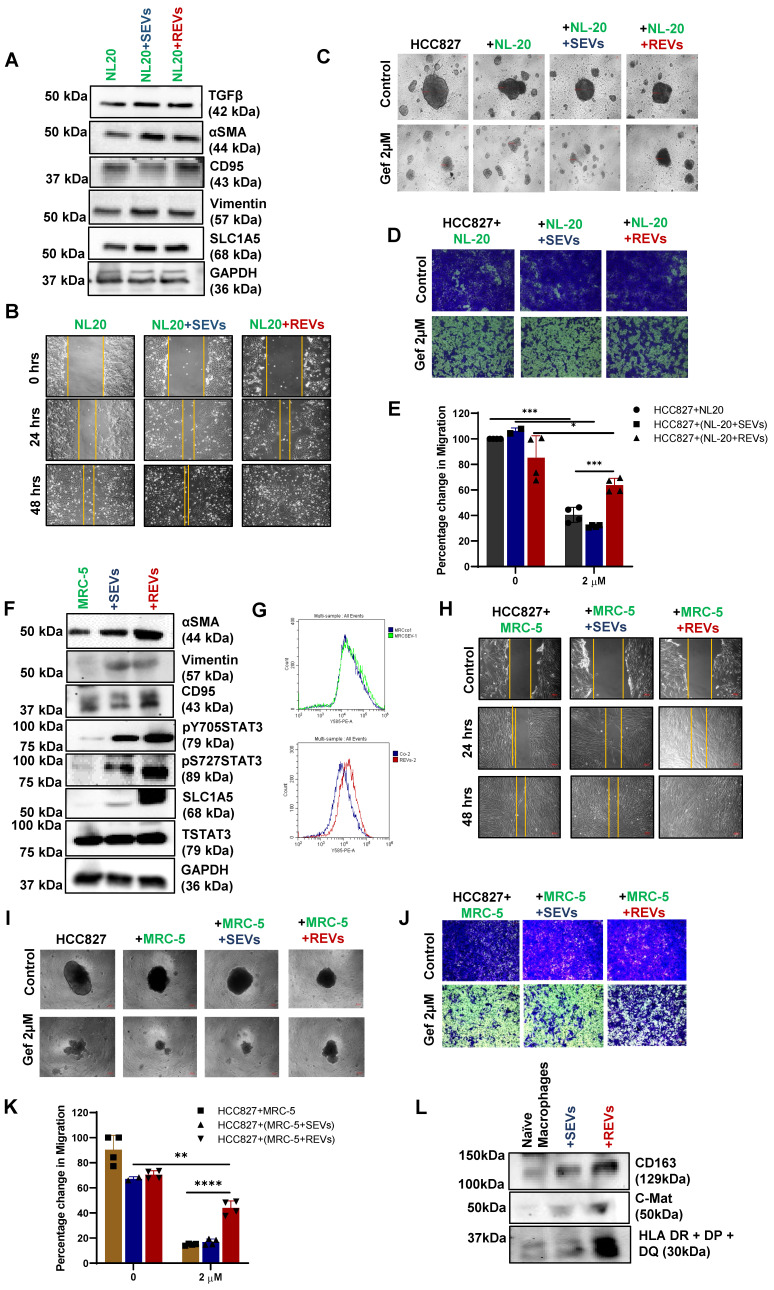
** REVs regulate tumor microenvironment. (A)** Western blot showing increased SLC1A5, TGFβ, αSMA, CD95, and Vimentin levels in total lysates of NL-20 cells exposed for 48 hours with SEVs and REVs. GAPDH was used as a loading control. **(B)** The scratch assay shows an increase in the migratory capacity of NL-20 cells exposed to SEVs and REVs for 48 hours (Scale bar: 10 cm). **(C)** Effect on spheroid formation was assessed in the co-culture (NL-20, NL-20+SEVs, and NL-20+REVs with HCC827) cells after treatment with gefitinib for 24 hours; 3000 cells were re-seeded on low attachment spheroid plates and left for 7-10 days before viewing under the microscope using 10X magnification (Scale bar: 10 mM).** (D)** From the same setup, 75,000 cells were re-seeded in ThinCert® cell culture inserts for 48 hours, stained with crystal violet, and viewed under a microscope (Scale bar: 100 µm) and **(E)** quantified by dissolving with 33% (v:v) acetic acid and read at an absorbance of 590 nm as described in Materials and Methods. Migration rates are plotted in percentages with respect to control cells. **(F)** Western blot showing increased SLC1A5, αSMA, CD95, Vimentin, STAT3pY705, and STAT3pS727 levels in total lysates MRC-5 cells exposed for 48 hours with SEVs and REVs. Total STAT3 and GAPDH was used as loading controls. **(G)** Increase expression of FAP in MRC-5 cells exposed to REVs and analyzed using Flow cytometry. For flow analysis, at least 10,000 cells were analyzed as described in Materials and Methods. (H) Scratch assay shows an increase in the migratory capacity of NL-20 cells after 48 hours of exposure to SEVs and REVs. **(I)** Effect on spheroid formation was assessed in the co-culture (NL-20, NL-20+SEVs, and NL-20+REVs with HCC827) cells after treatment with gefitinib for 24 hours; 3000 cells were re-seeded on low attachment spheroid plates and left for 7-10 days before viewing under the microscope using 10X magnification (Scale bar: 10 mM). **(J)** From the same setup, 75,000 cells were reseeded in ThinCert® cell culture inserts for 48 hours stained with crystal violet and viewed under a microscope (Scale bar: 50 µm) and **(K)** quantified by dissolving with 33% (v:v) acetic acid and measuring absorbance at 590 nm as described in Materials and Methods. Migration rates are plotted in percentages with respect to control cells. Data are representative of at least 3 independent experiments and shown as mean ± SD of biological triplicates. Two-way ANOVA was employed for statistical significance (*p < 0.05, **p < 0.01, ***p < 0.001, ****p < 0.0001). **(L)** Western blot showing increased CD163, C-Mat, and HLA DR+DP+DQ level in naïve macrophages (M0) after being exposed to SEVs and REVs for 48 hours.

So far, we have found that EVs released from TKI-resistant tumor cells induce resistance to cancer therapy in many tumor types by upregulating glutamine transporter. EVs mediate communication between tumor cells and tumor microenvironment (TME) 19. Two major components of TME are cancer-associated fibroblasts (CAFs) and tumor-associated macrophages (TAMs) 20, which are programmed to support cancer cell invasion and metastasis. First, exposure of normal lung epithelial cell line (NL-20) to REVs and SEVs for 48 hours resulted in increased expression of SLC1A5, EMT marker Vimentin, and other tumor markers (Figure 6A). Enhanced migration of cells was seen as well after 48 hours of exposure (Figure 6B). Next, we co-cultured NL-20 cells pre-exposed to REVs or SEVs for 24 hours with HCC827 cells for 4 days (Figure S6A) followed by treatment with gefitinib for 24 hours. The resulting spheroid formation and migration assays' data show no effect of gefitinib on HCC827 cells co-cultured with NL-20+REVs (Figure 6C- E).

We also used another approach for co-culture where we plated HCC827 cells in the bottom well and NL-20 cells with or without SEVs and REVs exposure in the insert (Figure S6B) for 48 hours followed by gefitinib treatment for 24 hours. The resulting data revealed no significant difference in spheroid formation, whereas, migration assays showed significant blunting of the effect of gefitinib in cells treated with REVs (Figure S6C- E). The experiment was repeated with a fibroblast cell line (MRC-5) to determine the effects of REVs on stromal fibroblasts. REVs were found to enhance the expression of CAFs related proteins like αSMA, CD95, EMT marker vimentin, SLC1A5, and STAT3 phosphorylation as well as FAP expression after 48 hours exposure to REVs and SEVs (Figure 6F-G) suggesting that REVs can subvert stromal fibroblasts to adopt a cancer-promoting phenotype. Scratch and co-culture of HCC827 with MRC-5 also support our earlier observation with NL-20 cells (Figure 6h-K and S6F- H).

Cancer immune evasion is known to be associated with polarization of macrophages from an M1 to M2 phenotype. An increase in STAT3 activation has been shown as the primary signaling of macrophage polarization 21. To check the effect of TKI-resistant EVs on macrophages, we generated M0 macrophages using U937 cells after exposure to 10 ng/ml of PMA (Figure S6I). Protein expression data reveal the activation of macrophages with REVs compared to SEVs (Figure 6L).

**Figure 7:**
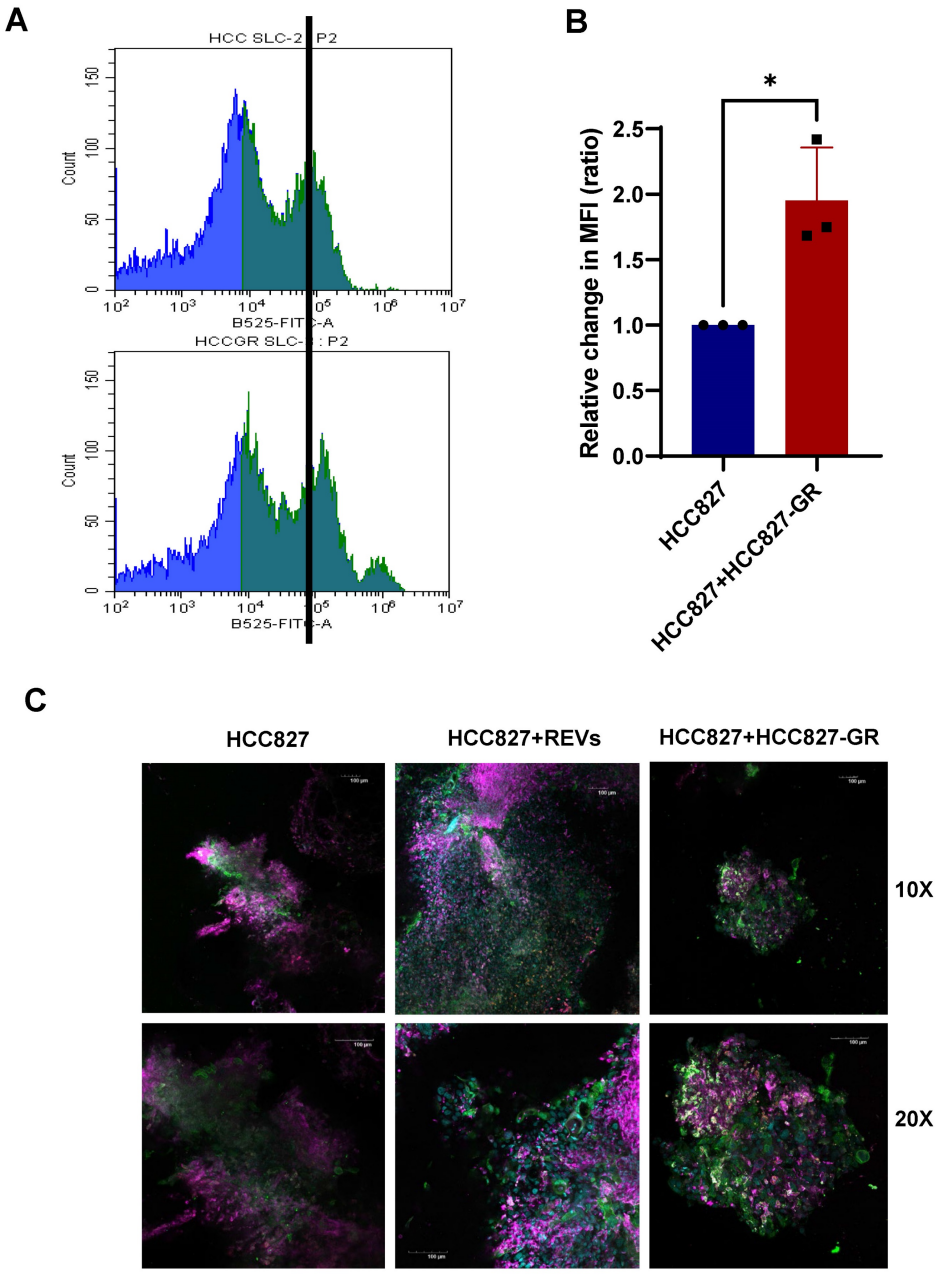
** REVs regulate tumor microenvironment *in vivo*. (A, B)** Flow cytometry data showing increased expression of SLC1A5 on HCC827 xenograft slices 48 hours after co-culture with HCC827-GR slices. For flow analysis, at least 10,000 cells were analyzed by flow cytometry as described in Materials and Methods (MFI: Median fluorescence intensity). Data are representative of at least 3 independent experiments and shown as mean ± SD of biological triplicates. An unpaired T-test was employed for statistical significance (*p < 0.05).** (C)** Surface expression of SLC1A5, CD163, and FAP was assessed following co-culture of HCC827 xenograft slices with HCC827-GR xenograft slices for 48 hours. Image viewed at 10X and 20X magnification by confocal microscopy (Scale bar: 100 µM).

To further validate these *in vitro* findings, we set up xenograft murine models of HCC827 and HCC827-GR cell lines. Murine-derived tumor slices from the individual models (HCC827 and HCC827-GR) were either cultured alone or co-cultured. Slices from HCC827 xenografts were exposed to SEVs and REVs for 48 hours before analyses by flow cytometry and confocal microscopy. Results show increased expression of SLC1A5 and FAP by flow cytometry and confocal imaging (Figure S7A-D), and CD163 (confocal imaging) in HCC827 tumor slices incubated with REVs or when co-cultured with HCC827-GR tumor slices (Figure 7A-C).

Collectively, these results signify that EVs from TKI-resistant tumors, added exogenously or secreted by resistant tumors during co-culturing, are involved in reprograming the tumor microenvironment, thereby promoting tumor progression and resistance.

## Discussion

Resistance to molecular targeted pathway inhibitors arise through many factors such as acquisition of mutations in the target protein, activation of alternative signaling pathways 22, intratumor heterogeneity 23. Previous studies have linked EVs with TKI/drug resistance 24-26 by way of EVs from resistant cells transferring resistant phenotype to sensitive cells in many type of tumors 27,28. Nevertheless, the mechanism of how cells interact to induce therapeutic resistance remains unclear. In this manuscript we describe a mechanistic pathway whereby treatment resistant tumour cells convey this resistant phenotype by releasing REVs that are taken up by sensitive cells. These REVs not only affect tumor cells; they can also transform non-malignant cells in the TME to support tumorigenesis. Analysis of EVs of TKI-resistant cancer cells as well as plasma of patients with TKI-resistant metastatic cancer reveal an enrichment in SLC1A5 and SLC25A5 solute carrier transporters and ALDHA1. Of clinical relevance, patients with NSCLC with higher SLC1A5 in plasma EVs are more resistant to treatment as evidenced by shorter PFS. We further demonstrated that cells that are incubated with REVs upregulate expression of these proteins and demonstrate metabolic shift to oxidative phosphorylation (OXPHOS) as well as STAT3 activation and are rendered more resistant to their respective TKIs, which is consistent with the association of OXPHOS and TKI resistance in our previous work 3. To validate this clinically, we showed that EVs derived from primary tumour cell line of a patient with secondary resistance to osimertinib could confer resistance an osimertinib sensitive EGFR T790M positive cell line. Mechanistically, SLC1A5 was potentially incorporated in the cell membrane of recipient cells, and functionally increased glutamine uptake to drive oxidative phosphorylation.

SLC1A5 is a member of the solute family of membrane bound transporters and is expressed in cancer cells where it facilitates influx of glutamine to supply proliferative cells with the required bioenergetic substrates for metabolic and signalling functions 29. SLC1A5, in particular, shown to be upregulated in lung cancer 16, breast cancer 30, head and neck cancer 31, and colorectal cancer 32. Additionally, a recent report has revealed that a variant of SLC1A5, located in the inner mitochondrial membrane, plays a crucial role in metabolic reprogramming and mitochondrial glutamine metabolism in pancreatic cancer 17.

Glutamine has been shown to shift cancer cells towards utilising oxidative phosphorylation for bioenergetics and/or activate STAT3 to promote tumor progression 33-35. It is well established that cancer cells are highly dependent on glutamine to fuel the mitochondrial tricarboxylic acid cycle and to provide the carbon backbone for synthesis of critical macromolecules like nucleotides and fatty acids. Its role in the immune compartment of the tumor microenvironment is actively being studied. 36. Cumulatively, these studies provide substantial evidence highlighting the pivotal role of glutamine metabolism in cancer metastasis and therapy resistance.

TDEs have been known to transfer oncogenic molecules to modulate tumor phenotype. For example, glioma cells expressing EGFR variant III (EGFRvIII) secrete these in EVs that are internalised by EGFRvIII negative recipient cell in the tumor to activate MAPkinase and protein kinase B signaling pathways and promote tumor phenotype 37. Concordant with our findings demonstrating treatment resistance through transfer of membrane bound transporters on REVs, transfer of functionally active membrane bound proteins between cells to redirect cell function is established. An example is in prion mediated neurodegenerative disorder mediated where exosomes convey abnormally folded prion protein (PrP) scrapie (PrPsc) on their membrane to other neural cells leading to the accumulation of these prion proteins in the central nervous system 38,39. Furthermore, membrane transporters carried on EVs have been shown to be correctly transferred and functional after cellular transfer 40.

Whilst the transfer of TDEs between cancer cells leading to resistance is an important step in evolution of cancer, the effects of these TDEs on non-malignant cells within the tumour microenvironment leads to further subversion of cells to promote tumour growth. Utilising tumor tissue slices *ex vivo*, we showed that transfer of REVs derived from cancer cells enhances the motility and invasiveness, expression of EMT genes in epithelial cells and fibroblasts and polarises macrophages to immunosuppressive phenotypes more than SEVs. The mechanism through which REVs confer these characteristics to non-malignant cells is unknown but warrants further investigation. Immune evasion of cancers is a complex process involving multiple mechanisms. TDEs can promote immunosuppressive macrophage M2 polarisation via nucleic acid payloads like miRNA, lncRNA and circular RNA 41,42. Glutamine catabolism contributes in macrophage activation as well as glutamine also support IL4-induced macrophage polarization 43. Fatty acids carried by TDEs to dendritic cells to activate PPARγa to utilise OXPHOS from glycolysis suppressing DC function 44. PDL1 carried on the membrane of circulating EVs from malignant melanoma have been shown to be associated with resistance to anti-PD1 axis blockade through immunosuppressive effects 9.

Despite the strong support for the role of REVs in promoting treatment resistance through intercellular transfer of proteins, limitations in this study include the fact that EVs harvested from cells *in vitro* for incubation experiments may be too concentrated and non-physiological. In our experiments we have made every effort to utilise concentrations of TDEs that we believe are physiological and circulating in patients with metastatic cancers, increasing the plausibility that cancers utilise this circulating form of cell-cell communication to promote drug resistance.

Clinically, analysis of TDEs in circulation for an increase in SLC1A5 and SLC25A5 could potentially indicate the development of treatment resistance, particularly in the context of TKI resistance. Further studies to assess this would be helpful to determine the clinical utility. The involvement of this SLC1A5-glutamine axis also supports the development of therapeutic interventions against this preferentially upregulated pathway in cancer and in this regard SLC1A5 and glutaminase inhibitors are already in experimental development.

## Conclusion

In summary, our study reveals that EVs derived from TKI-resistant cells induce resistance in sensitive cells by modulating the metabolic profile of the tumor microenvironment. We conclude that EVs from resistant cells promote cell migration and proliferation by upregulating glutamine transport, particularly SLC1A5, which enhances OXPHOS and therapy resistance. These results have the potential to inspire the development of therapeutic strategies targeting metabolic reprogramming in resistant cells. Inhibitors of SLC1A5 could be developed and utilized in combination with TKIs to effectively combat therapy resistance.

## Material and Methods

### Patient and sample collection

Plasma samples from 111 patients with TKI-resistant NSCLC, 1 breast cancer, I ovarian cancer, 1 colon cancer and 58 healthy volunteers were collected after taking their consent at Cancer Science Institute and National University Hospital. Collected plasma was stored at -80 ^0^C till EVs isolation.

### Cell culture and transfection

H1975 (ATCC), HCC827 (ATCC), HCC827-GR, U937 (ATCC), and NL-20 (ATCC) cells were cultured in RPMI-1640 media (Hyclone), while A375 (ATCC), A375-VR were cultured in DMEM high glucose media (Hyclone); MRC-5 (ATCC) were cultured in EMEM (Sigma); all the media were supplemented with 10% fetal bovine serum (FBS), 1% penicillin-streptomycin and 1% L-glutamine. To maintain resistance to their respective drugs, cells were continuously cultured with 2 μM gefitinib (HCC827-GR), 2 μM vemurafenib (A375-VR). All cells were kept at 37 °C incubator with 5% CO_2_.

For knockdown of *SLC1A5* and *SLC25A5* (Horizon Discovery Ltd), SMARTpool siRNA was transfected into HCC827-GR cells using Dharmafect transfection reagent 1 (Dharmacon, USA) for 24 hours. Overexpression of pcMV6-SLC1A5-GFP was done using Lipofectamine ® LTX with PLUS^TM^ reagent (Thermo Fisher Scientific, USA) for 48 hours according to manufacturer's protocol. After transfection GFP cells were sorted and single cell were grown to make a stable cell line using G418 (Geneticin) for selection.

### Chemical and reagents

For SLC1A5 knockdown using siRNA, following targeting ON-TARGETplus human *SLC1A5* SMARTpool siRNA (set of 4 siRNA sequences Dharmacon, USA) were used: 1. GCCUUUCGCUCAUACUCUA, 2. GGUCGACCAUAUCUCCUUG, 3. GCAAGGAGGUGCUCGAUUC, 4. UGAUACAAGUGAAGAGUGA.

For SLC25A5 knockdown using siRNA, following targeting ON-TARGETplus human *SLC25A5* SMARTpool siRNA (set of 4 siRNA sequences Dharmacon, USA) were used: 1. GAAGAUUGCUCGUGAUGAA, 2. CACCCAGGCUCUUAACUUC, 3. GCUCUACUUUGCAGGGAAU, 4. ACGUGUCUGUGCAGGGUAU. The antibodies used for immunoblotting: SLC1A5, SLC25A5, S727STAT3, and Total STAT3 (Cell signaling, USA), β-actin and c-Met (Santa-Cruz, Dallas, TX), ALDH1A1, Y705STAT3, CD63, CD81, TSG101, CD9, Anti-HLA DR + DP + DQ, and Calnexin (Abcam, Cambridge, UK), CD44, CD24, CD133 (BD Bioscience), CD163 (ThermoFisher Scientific, USA), Anti-SLC1A5-FITC antibody were purchased from Alomone labs and Anti-FAP from R&D systems. GPNA, V9302 and STATIC were purchased from Sigma. Anti-mouse Alexa flour 647 antibody was purchased from Molecular probe.

### EVs isolation from cell line and human plasma samples

80-90% confluent cells were washed first with 1XPBS (HyClone, USA) for 3 times and followed by wash twice with plain RPMI. After washing, cells were incubated with RPMI containing 5% exosomes-free FBS for 24 hours. Culture medium were collected and centrifuged at 2000g for 10 minutes to remove cells, followed by centrifugation at 4500rpm for 10 minutes in Amicon ultra 15 ml 0.5 filter (Millipore, USA). Resultant concentrated supernatant were diluted with 1XPBS and then centrifugated at 100,000g for 1.5 hours at 4 °C. The EV pellets were washed with 1XPBS at the same speed and pellets were dissolved in 1XPBS. Total Exosome Isolation Reagent (Invitrogen, USA) was used to isolate EVs from transfected cells and patient-derived plasma according to manufacturer's protocol. Briefly, after first centrifugation, culture media were incubated with transfection reagent for overnight at 4 °C. Then precipitated exosomes were centrifugated at 10,000g for 1 hr at 4 °C and resultant EV pellets were dissolved in 1XPBS or 1X RIPA lysis buffer.

Isolation of EVs from patient-derived plasma was done by ultracentrifugation as described earlier. Briefly 500µl plasma was thawed on ice and first centrifuged at 2000g for 20 minutes to remove debris. Next, plasma were diluted with 9.5 ml of 1XPBS and centrifuged at 100,000g for 1.5 hr at 4 °C. Pellets were washed with 1XPBS at the same speed and dissolved in 100µl of 1XPBS. For ELISA from plasma exosomes, total exosome isolation kit (Invitrogen, USA) was used. Briefly, after removing debris from plasma, plasma was incubated with 0.5 volume of 1XPBS and 0.2 volume of exosome precipitation reagent for 10 minutes. Exosomes were dissolved with 100 µl of 1XPBS or 1X RIPA Lysis buffer after centrifugation at 10,000 g for 5 minutes at 4 °C. Protein estimation was done using Coomassie Plus (Bradford) Protein Assay (Thermo Fisher Scientific, USA). The isolated EVs were stored in -80 °C till further characterization, analysis or experimental use.

### TMT labeling and proteomics analysis

Tandem mass tag (TMT)-based quantitative liquid chromatography-mass spectrometry (LC-MS) analyses of EVs were performed at the National Technology University Proteomics Resource Center. EVs were lysed in 8 M urea (pH = 8.0 in PBS) with 1 mM PMSF, 1 mM proteinase inhibitor cocktail 3 (Sigma-Aldrich, Missouri, MO, USA), and the protein concentration of each sample was measured using a BCA kit (Thermo Scientific, Waltham, MA, USA) according to the manufacturer's instructions. The protein solution (50 μg) of each sample was reduced, alkylated, acetone-precipitated, resuspended, digested, desalted, and labeled with TMT (Thermo Scientific). In brief, the raw MS data to identify and quantify peptides and proteins were analyzed, and MS/MS spectra were searched against the UniProtKB Homo sapiens database. For analysis we used the following criteria for stringent cut-off, Filter 1: FDR ≤ 1%, 99% confidence. Filter 2: Peptide identification ≥ 2 per quantified protein., and Filter 3: Protein quantified in at least 2 out of 3 replicates. For upregulation fold change > 1.2 and downregulation fold change < 0.8.

### PKH67 and Acoerela staining

Isolated EVs were diluted in diluent C according to the manufacturer's instructions. Prior to staining, 2x dye (PKH67, Green Fluorescent Cell linker for General Cell Membrane, Sigma-Aldrich) solution was prepared and added to the diluted EVs. After 5 minutes incubation staining was stopped using 2 ml serum for 1 minute. For Acoerela staining, Isolated EVs were diluted in 1ml of 1XBS and then diluted Aco-600 (NUS) added as 2.5 µM final conc. After 1 h of staining 1ml 1XPBS was added. To remove unbound dyes, the stained EVs were ultracentrifuged (Optima MAX-XP, Beckman Coulter) at 100,000g for 60 minutes at 4 °C (TLA 90 rotor). EVs were washed with plain media and added to the cell culture for internalization before imaging using the FV3000 OLYMPUS microscope (Tokyo, Japan).

### Enzyme-linked immunosorbent assays (ELISA)

Cell culture media of target cells were collected into microcentrifuge tubes and spun down at 1500 rpm for 10 minutes at 4 °C to remove any cellular debris. The resulting supernatant was transferred to a new tube and subjected to the manufacturer's instructions of the Human IL-6 ELISA Kit (Thermo Fisher Scientific, USA). Lysates of plasma EVs were made and ELISA was done according to manufacturer's instruction of SLC1A5, SLC25A5 and ALDH1A1 ELISA kit (My BioSource Inc, USA).

### Western blotting

Cells or EVs were lysed in RIPA lysis buffer containing PMSF and phosphatase inhibitor cocktail 3 (Sigma-Aldrich, USA). The protein concentration of each sample was measured using a BCA protein assay kit (Thermo Fisher Scientific, USA). Equal amounts of the protein samples (30 μg per sample) were separated on an 8% or 10% sodium dodecyl sulfate-polyacrylamide gel electrophoresis (SDS-PAGE) followed by transfer onto an immuno-Blot PVDF membrane (Bio-Rad, Hercules, CA, USA) using wet transfer method. After blocking the membrane with 5% milk or casein in TBS with 0.1% Tween-20, the blots were incubated with primary antibodies overnight at 4 °C on the shaker and probed with appropriate secondary antibodies conjugated with horseradish peroxidase (HRP). After washing with TBS+ 0.1% Tween 20 membranes were exposed to EZ-ECL substrate Western Blotting Kit (Pierce, Rockford, IL, USA) and signals were detected using iBright Chemidoc (Thermo Fisher Scientific, USA).

### Crystal violet, colony, spheroid formation, and matrigel assay

Approximately 2-3x10^5^ cells were seeded onto 6-well plates. The next day, different concentrations of EVs were added to the plate for 24 hours and then cells were re-pated for cell viability assay, colony formation, and spheroid formation. Subsequently, Gefitinib, Osimertinib, Crizotinib, Defactinib, Selumetinib, Afatinib, Bosutinib, and Debrafenib (H1975, HCC827) or Vemurafenib (A375) were added for 48 hours. The treated cells were collected via trypsinization and re-seeded at approximately 3000 cells/well in either 6-well plates (2D Colony Formation Assay) or Corning® Spheroids 96-Well Microplates (Tumor Spheroids Formation Assay). Spheroids and colonies formed after 6 and 14 days respectively were imaged using the ZEISS® Axio Vert (Oberkochen, Germany). A1 Microscope. For Matrigel assay, a small spheroid was formed through the hanging drop method. Briefly, 500-1000 cells/25 µl 10-15 droplets were dropped on the cover of a 10 cm culture plate with 5 ml 1XPBS in the bottom plate and let spheroid grow in the incubator. After 72 hours spheroids were collected, mixed with 1:1 diluted matrigel, and placed on a preset 2.5% Matrigel 24 well plate. Culture media was added on top of the spheroid and incubated for 48-72 hours before Imaging at ZEISS® Axio Vert (Oberkochen, Germany). For confocal microscopy, the Spheroid was fixed using 2% paraformaldehyde followed by permeabilizing with 1% NP40. DAPI was added before imaging using the FV3000 OLYMPUS microscope.

### OCR measurement

Cells were plated at respective optimal densities in Seahorse XF 24-well plates one or two days prior to the measurement and incubated in Seahorse XF Assay Media at 37 °C for 1 h without CO_2_ just before running the assay. Substrate concentrations were 1 µM for Oligo and FCCP, and 1 µM/0.5 µM for Rot/AA. All the reagents for OCR were purchased from Seahorse Bioscience. OCR measurements were obtained using the Seahorse XFe24 Analyzer (Agilant, USA), and normalized to protein concentration (µg/µL).

### Flow cytometry

Cells were washed with 1XPBS +1%BSA before staining. For staining, cells were incubated with SLC1A5-FITC 1mg/ml (Alomone labs, Israel) and FAP-PE (R&D systems, USA) 10 µl/10^6^ cells for 1h. To stain cells from tumor slices, single cell suspension was obtained using a Human tumor dissociation kit (Milteny Biotec, USA) and then stained with SLC1A5-FITC 1mg/ml and FAP-PE 10 µl/10^6^cells for 1h. Cells were analyzed by CYTOFLEX LX flow cytometer (Beckman Coulter, Indianapolis, USA). Cells were loaded with MitoSox™ RED MITOCHODNRIAL O_2_^-^ INDICATOR (Molecular Probes, Invitrogen Corporation, USA) for mitochondrial O_2_^-^, and 3,3'-Dihexyloxacarbocyanine Iodide (DiOC6, Invitrogen, USA) for mitochondrial transmembrane potential as described previously 45 and analyzed by FACS caliber flow cytometer (BD LSR Fortessa, USA). At least 10,000 events were recorded and then plotted using CytExpert 2.1.

### Transwell migration assay

Cell migration was assessed by measuring the rate of movement of cells across a porous membrane towards a chemoattractant within a specified time using a transwell cell migration assay. HCC827-pcmv6 (Addgene, Singapore) and HCC827-SLC1A5-OE (Addgene, Singapore) cells treated with gefitinib for 24 hours and co-cultured HCC827 with NL-20 or MRC5 cells grown were trypsinized, washed in PBS, and resuspended in serum-free RPMI medium to obtain a final concentration of about 5 x 10^5^ cells/ml. The 24-well ThinCert^®^ cell culture inserts (Greiner Bio-One GmbH, 662638) with 8-μm pores and translucent PET membranes were placed in the wells of an empty CELLSTAR® 24-well cell culture plate (Greiner Bio-One GmbH, 662638). Cell suspension (150 µl, 75,000 cells) was added into the cell inserts and 600 µl of 10 % FBS McCoy 5A (modified) medium was carefully added into the wells holding the inserts to create the chemoattractant gradient. Inserts with the plates were left in CO_2_ incubator for 48 hours. Cotton bud tips were used to gently scrape and remove any cells on the inner chamber of inserts that had not migrated. Inserts were then carefully dipped and fixed with crystal violet solution, washed twice with distilled water and dried before images of the migratory cells on the outer PET membranes were viewed under ZEISS^®^ Axio Vert. A1 microscope (Oberkochen, Germany) at 10 X magnification. The stained and bound migratory cells were also quantified by dissolving and eluting the crystal violet in 400 µl of 33 % (v:v) acetic acid for 20 min on a shaker. The absorbance reading (590 nm wavelength) of the eluted crystal violet solution was measured using TECAN GENius PLUS microplate reader.

### RNA sequencing analysis

Total RNA extraction was carried out using Trizol (Life Technologies, Carlsbad, CA, USA). RNA purity and integrity were assessed using a NanoDrop 2000 spectrophotometer (NanoDrop Technologies, Wilmington, DE, USA). Total RNA (1 μg) was used for sequencing by Agilent technologies.

### RNA extraction and RT-qPCR

Total RNA was extracted using Trizol (Thermo Fisher Scientific, Waltham, USA) and reversely transcribed to cDNA with a cDNA Synthesis Kit (Qiagen, USA). The cDNA was subjected to real-time qPCR analysis with the Thunderbird SYBR qPCR Mix (Thermo Fisher Scientific) on a QuantStudio5 Real-Time PCR System (Thermo Fisher Scientific). The primer sequences synthesized by the Integrated DNA Technologies (Singapore) Relative gene expression was normalized to *ACTB* and calculated with the 2^-ΔΔCt^ method.

### Confocal microscopy and live imaging

HCC827 cells were plated in ibidi plates before incubation with FITC-conjugated SLC1A5 stained REVs. Imaging was performed using FV3000 OLYMPUS microscope (Tokyo, Japan) every 20 minutes from 4 hours till 36 hours after adding stained REVs.

For tissue imaging, tumor slices were cleaned from hydrogel and washed with 1XPBS. Slices were fixed with 4% formaldehyde for 2 hours at RT (room temperature) before blocking with 1% BSA + 1% FBS. After washing with 1XPBS for three times, slices were stained with FITC conjugated SLC1A5 (1:100 dilution), PE conjugated FAP antibodies (1:100 dilution) and CD163 (1:50 dilution) for 2 hours at RT. After washing with 1XPBS, anti-mouse Alexa flour 647 secondary antibody (1:1000 dilution) for CD163 was added for 12 hours with Hertz. After washing the slices with 1XPBS, cell clear was added and images were taken using FV3000 OLYMPUS microscope (Tokyo, Japan).

### Mouse xenograft studies and hydrogel embedding

Animal studies were conducted in accordance with protocol R23-0407 approved by the Institutional Animal Care and Use Committee (IACUC) at the National University of Singapore. BALB/c nude mice were used for the xenograft experiment. 5×10^6^ HCC827 TKI sensitive and resistant cells resuspended in 100 µl RPMI media were subcutaneously injected into both flanks of the mice. Mice were euthanized and tumors were excised at the end of the experiment.

Tumors were resected from mice and 300 µM slices were prepared as described in an earlier publication 46. After adding hydrogel solution into 6 well plates, MDTS (Mouse derived tumor slices) layering was done and supplemented with complete media with ROCK inhibitor (Y-27632, STEMCELL Technologies) on 1^st^ day and incubated at 37 ^0^C with 5% CO_2_ incubator. Slices of HCC827 were cultured with REVs and with slices of HCC827-GR for 2-3 days.

### Statistics

For all the experiments, two to three independent experiments were conducted and p-values were calculated using two-tailed paired or unpaired student's *t*-test and two-way ANOVA using GraphPad Prism software 9.0 (GraphPad Software Inc., San Diego, CA). P-values < 0.05 were considered statistically significant and all the P-values are mentioned in the figures and figure legends. Graphical abstract was illustrated using Biorender.

## Supplementary Material

Supplementary figures and tables.

## Abbreviations

EVs: Extracellular Vesicles
TKI: Tyrosine kinase inhibitor
MDTS: Mouse derived tumor slices
PFS: Progression-free survival
OCR: Oxygen consumption rate
OXPHOS: Oxidative phosphorylation
TME: Tumor microenvironment
TAM: Tumor associated macrophage
CAF: Cancer Associated fibroblast
NTA: Nanoparticle tracking analysis
PDCEV: Patient derived cell lines extracelluar vesicle
FAP: Fibroblast activation protein
