# Supplementary material for: Tumor-derived extracellular vesicles convey solute transporters to induce bioenergetic dependence shift contributing to treatment resistance
Source: Theranostics. 2024 Sep 30;14(16):6350–69. doi: 10.7150/thno.100374 (PMC11488098; doi:10.7150/thno.100374)
Supplement: Supplementary file 1 — Supplementary figures and tables. [file thnov14p6350s1.pdf]

Supplementary Figures:

Figure S1:

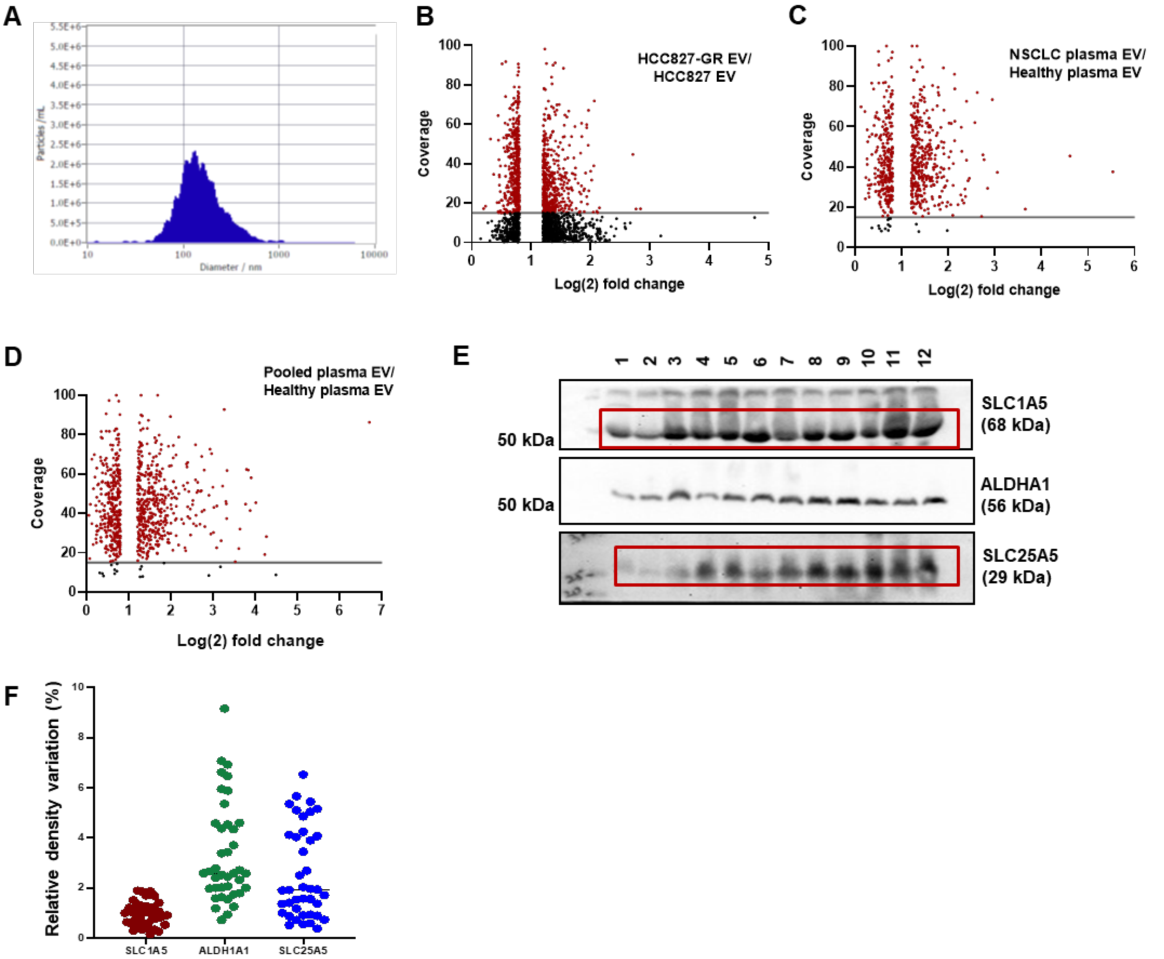

Figure S1: (A) NTA analysis was performed to obtain the size distribution of EVs isolated from plasma of patients with TKI-resistant tumors. **(B)** Volcano graph showing upregulation and downregulation of protein expression on EVs isolated from the supernatant of HCC827-GR cells in comparison with EVs from HCC827 cells (n = 3) and **(C)** EVs isolated from plasma of a patient with TKI-resistant NSCLC in comparison with EVs from plasma of healthy donor. **(D)** EVs were isolated from the pooled plasma of 5 patients with TKI-resistant tumors and compared to EVs from the plasma of healthy donor. **(E)** Western blot analysis of the expression of SLC1A5, SLC25A5, and ALDH1A1 in EVs isolated from the plasma of patients with TKI-resistant tumors (Lane 3 - 12) compared with EVs isolated from healthy donors (Lane 1, and 2). **(F)**

Expression analysis of SLC1A5, SLC25A5, and ALDH1A1 in EVs isolated from the plasma of patients with TKI-resistant tumors (N = 40) compared with EVs isolated from healthy donors (N = 20). Band expression values were derived using Image J software.

Figure S2:

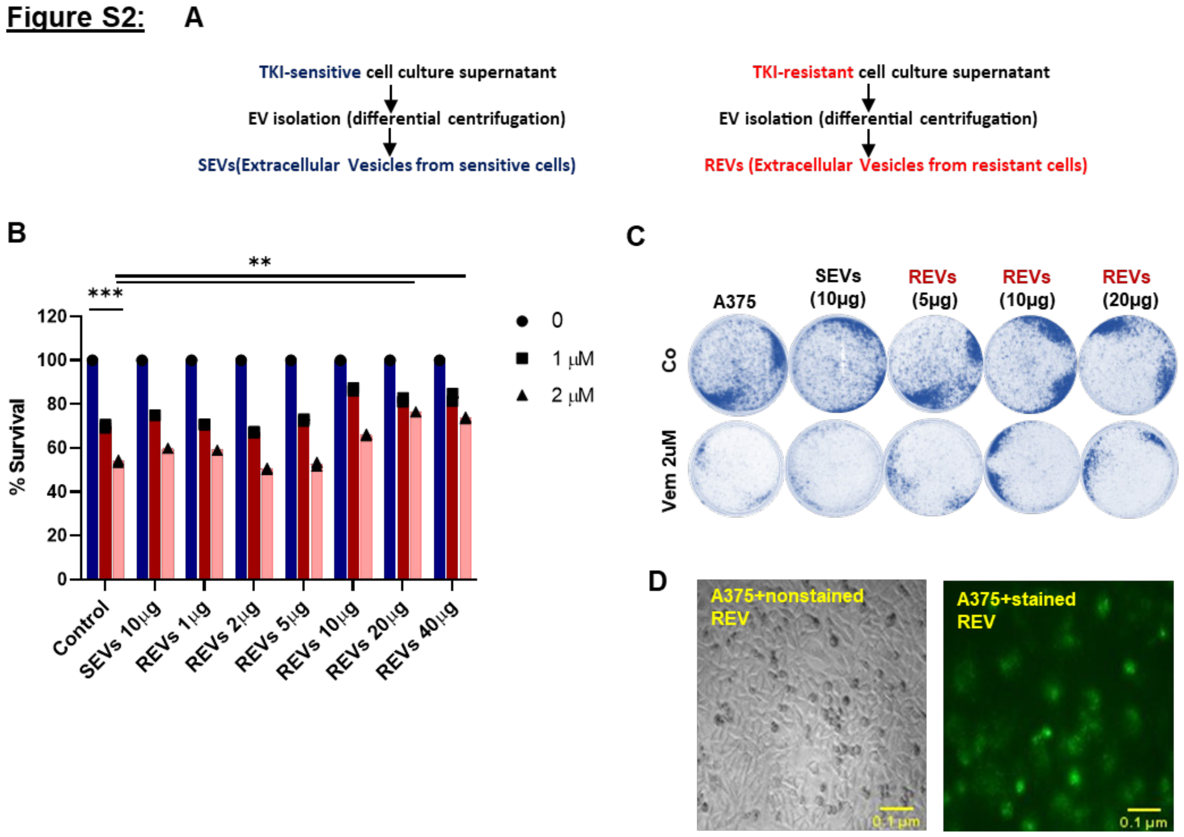

Figure S2: (A) Schematic illustration for the isolation of SEVs and REVs from TKI-sensitive (HCC827 or A375) and -resistant (HCC827-GR or A375-VR) cell lines **(B)** A375 cells were exposed to SEVs or increasing concentrations of REVs for 48 hours followed by gefitinib treatment for 24 hours and cell viability was measured by crystal violet staining. Data are representative of at least 3 independent experiments and shown as mean ± SD of biological triplicates. Two-way ANOVA was employed for statistical significance (**p < 0.01, ***p < 0.001). **(C)** Effects on long-term colony formation assessed in the samples from B; 3000 cells were re-seeded in 6-well plates and left for 7-10 days before staining with crystal violet or viewing under the microscope under 10X magnification. Results shown are representative of 3

independent experiments. **(D)** Isolated EVs from the supernatant of A375-VR cells were stained with PKH67 as described in Materials and Methods followed by incubation of A375 cells with the stained EVs and analyzed using confocal microscopy (Scale bar 0.1 μM).

Figure S3:

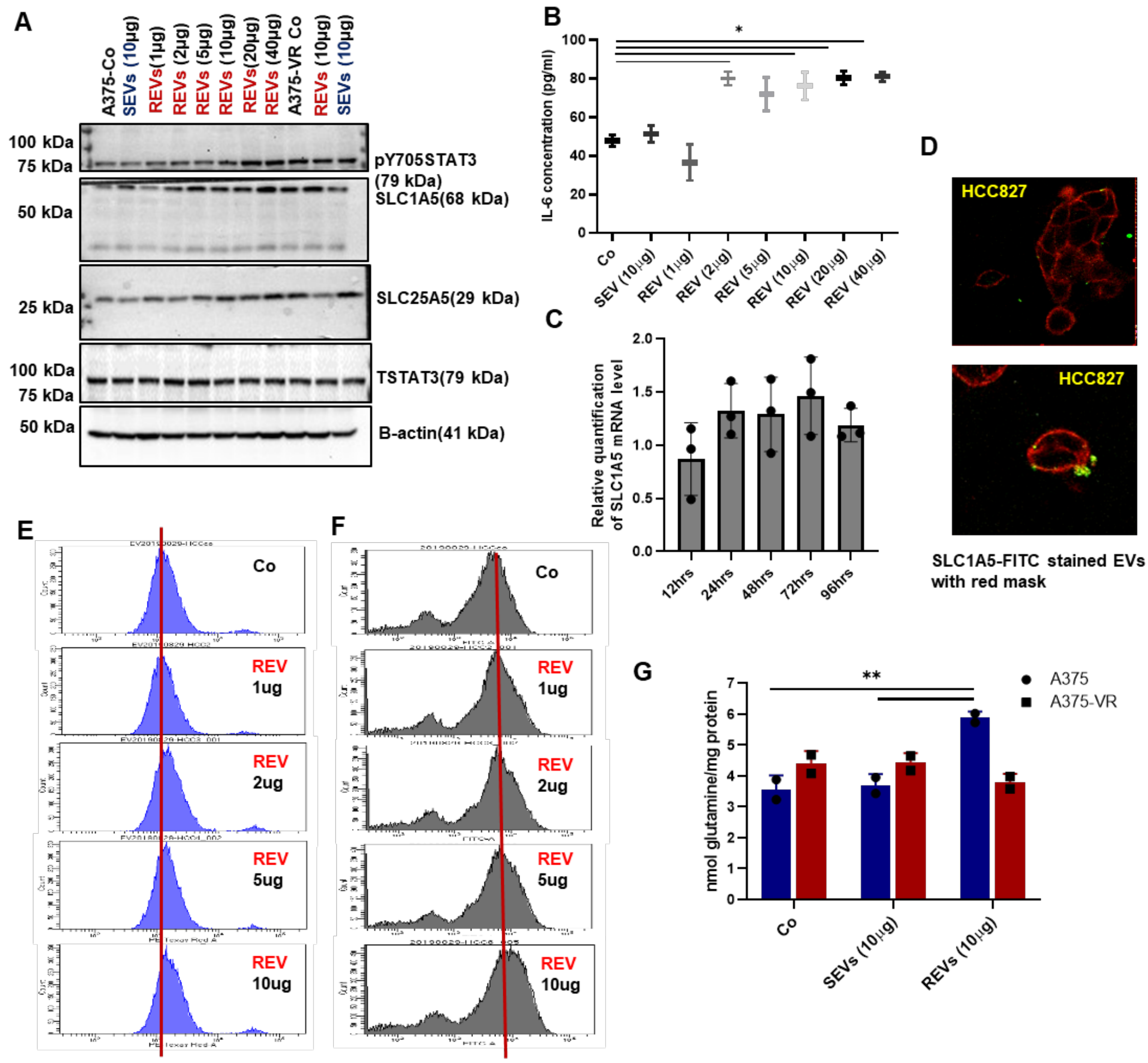

Figure S3: (A) Western blot analysis of SLC1A5, SLC25A5, and STAT3pY705 expression levels in whole cell lysates of A375 and A375-VR cells after 48 hours exposure to SEVs or increasing concentrations of REVs. Total STAT3 and β-actin were used as loading controls. **(B)** HCC827 cells were exposed to SEVs and increasing concentration of REVs for 48 hours and IL6 concentration was assessed in

the supernatant as described in Material and Methods. Data are shown as mean ± SD of biological triplicates and plotted using GraphPad prism software. Two-way ANOVA was employed for statistical significance (*$p < 0.05$). **(C)** HCC827 cells were exposed to 10 µg of REVs for the indicated durations and the *SLC1A5* transcript was assessed using SyberGreen as described in Materials and Methods. Data are representative of at least 3 independent experiments and shown as mean ± SD of biological triplicates. **(D)** SLC1A5-FITC stained REVs were added to HCC827 cells and live imaging was done using a confocal microscope from 4 to 36 hours for every 20 mins. **(E)** HCC827 cells were exposed to different concentrations of REVs for 48 hours, and stained with MitoSOX™ Red for detecting mitochondrial $O_2^{\cdot -}$ or **(F)** $DiOC_6$ for mitochondrial transmembrane potential. At least 10,000 cells were analyzed. Histogram data shown are representative of at least 3 independent experiments. **(G)** Intracellular glutamine levels in HCC827 cells after 48 hours of exposure to REVs. Glutamine was measured as described in Materials and Methods and plotted using GraphPad prism software. Data are representative of at least 2 - 3 independent experiments and shown as mean ± SD of biological triplicates. Two-way ANOVA was employed for statistical significance (**$p < 0.01$).

Figure S4:

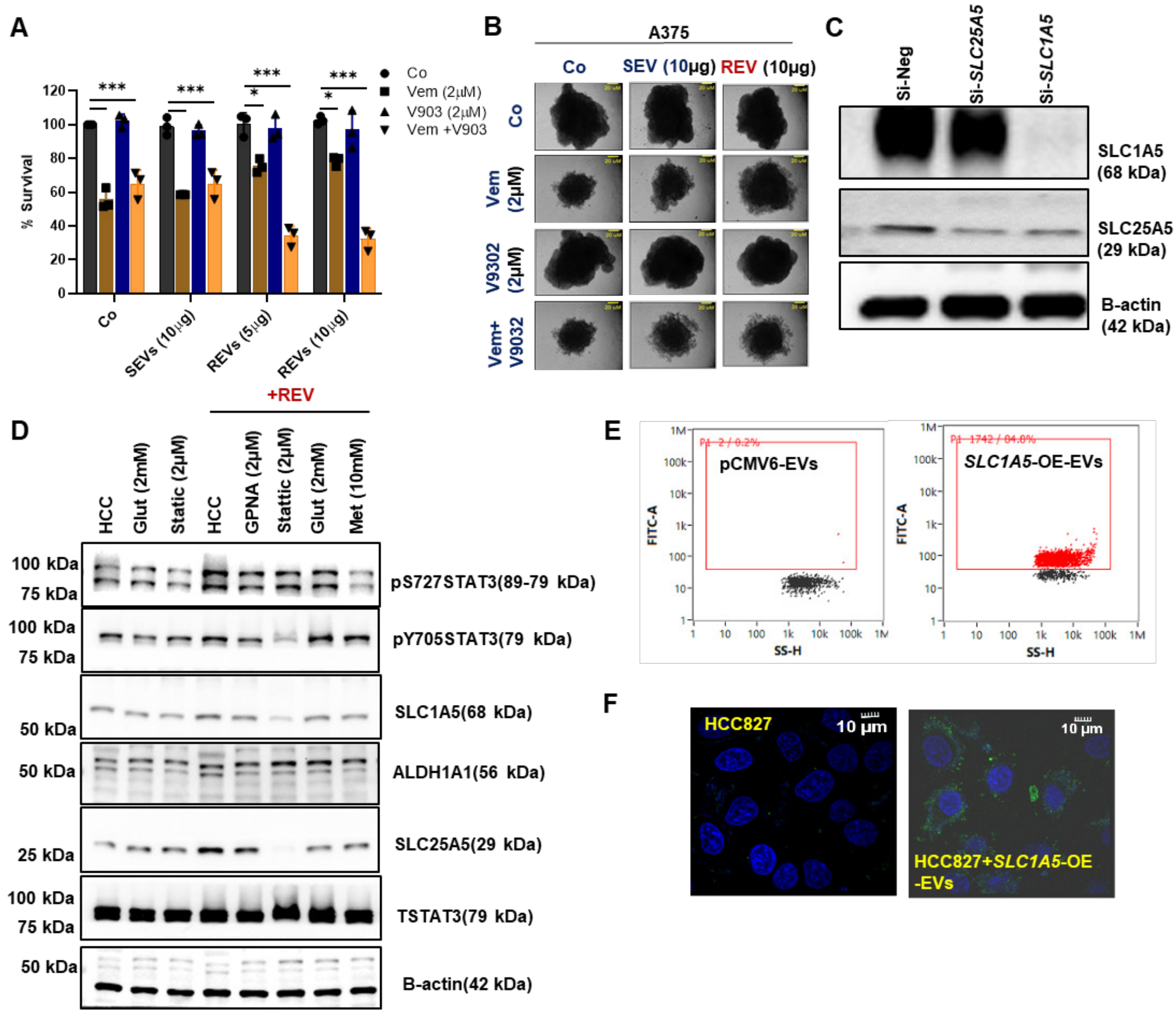

Figure S4: (A) A375 cells were pre-treated with REVs followed by incubation with V9302 (10 μM) for 1 h before exposure to Vemurafinib (2 μM) for 24 hours. Cell viability was measured using CCK-8 assay. Data are representative of at least 3 independent experiments and shown as mean ± SD of biological triplicates. Two-way ANOVA was employed for statistical significance (*p < 0.05, ***p < 0.001). **(B)** Effect on spheroid formation was assessed in the samples from A; 3000 cells were re-seeded on low attachment spheroid plates and left for 7-10 days before microscopy using 10X magnification (Scale bar: 20 μM). **(C)** Western blot analysis of the protein expression of SLC1A5 and SLC25A5 after siRNA-mediated gene silencing (Si*SLC1A5 or* Si*SLC25A5*) in HCC827-GR cells. **(D)** Western blot analysis of SLC1A5, SLC25A5,

STAT3pY705, and STAT3s727 levels in whole cell lysates of HCC827 cells exposed to REVs followed by treatment with GPNA and Static. Total STAT3 and β-actin were used as loading controls. **(E)** EVs were isolated from HCC827-*SLC1A5*-OE FITC-tagged cells and analysed by NanoFC. **(F)** Isolated EVs from HCC827-*SLC1A5*-OE FITC-tagged cells were added to HCC827 cells and images were captured using a confocal microscope (Scale bar 10 μM).

Figure S5:

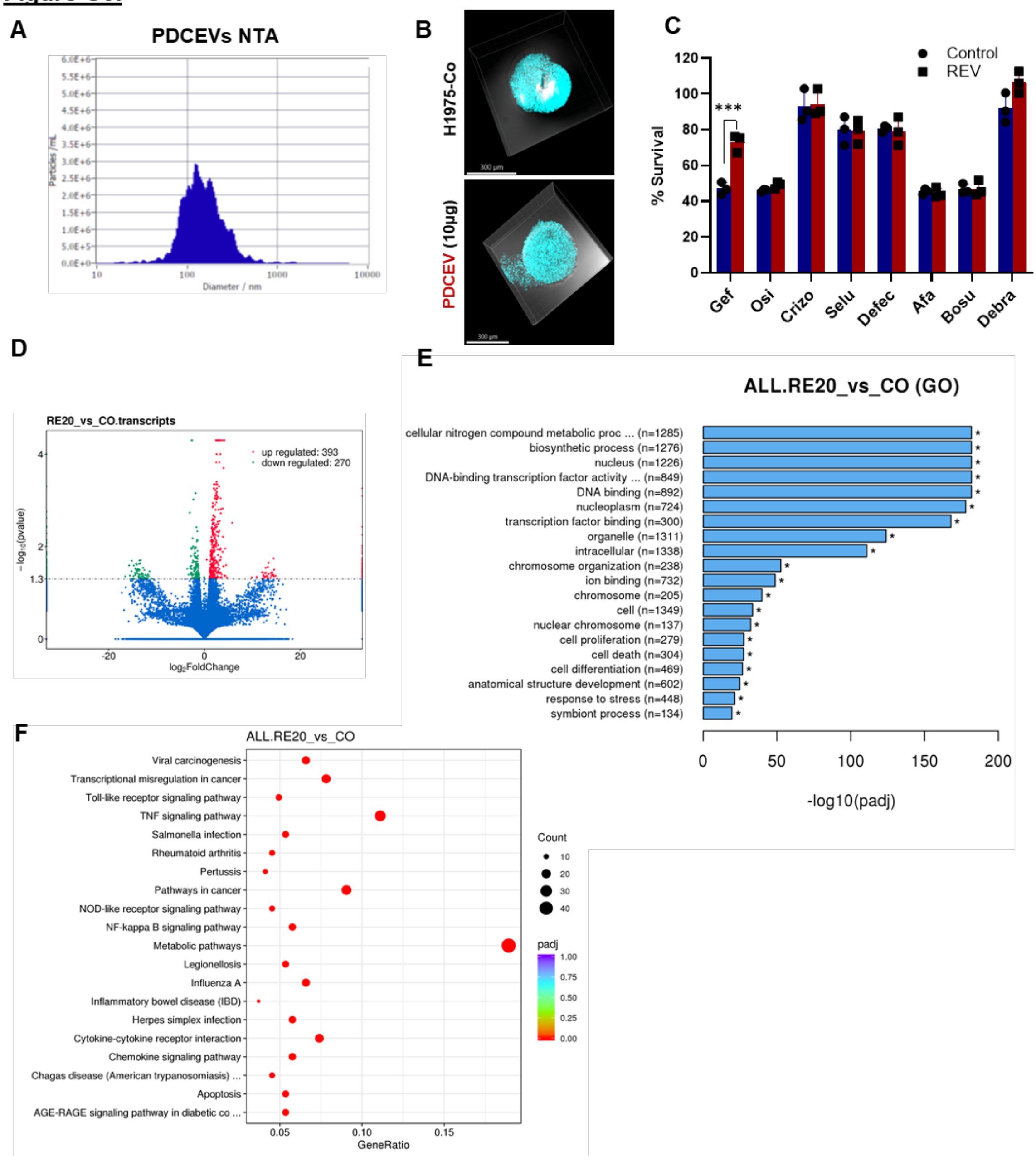

Figure S5: **(A)** NTA analysis showing the size distribution of EVs isolated from

Osimertinib-resistant patient-derived cell line. **(B)** H1975 cells with or without exposure to PDCEVs ( ) were assessed for spheroid formation using the hanging drop method and re-seeding in Matrigel. 7 - 10 days later, Z stack imaging was employed using confocal microscopy (Scale bar: 300 μM). **(C)** HCC827 cells were pre-exposed to REVs for 48 hours before incubation with 2 μM gefitinib, Osimertinib, Crizotinib, Selumetinib, Afatinib, Bosutinib, and Debrafenib for 24 hours. Cell viability was determined using CCK-8 assay. Data are representative of at least 3 independent experiments and shown as mean ± SD of biological triplicates. Two-way ANOVA was employed for statistical significance (***$p < 0.001$). **(D, E, F)** H1975 cells were exposed to 20 μg PDCEVs and expression analyses were performed against untreated H1975 cells. (D) Volcano graph showing upregulation and downregulation of transcripts **(E)** Gene Ontology data showing upregulation of transcripts or **(F)** KEGG data showing gene ratios for the various pathways.

Figure S6:

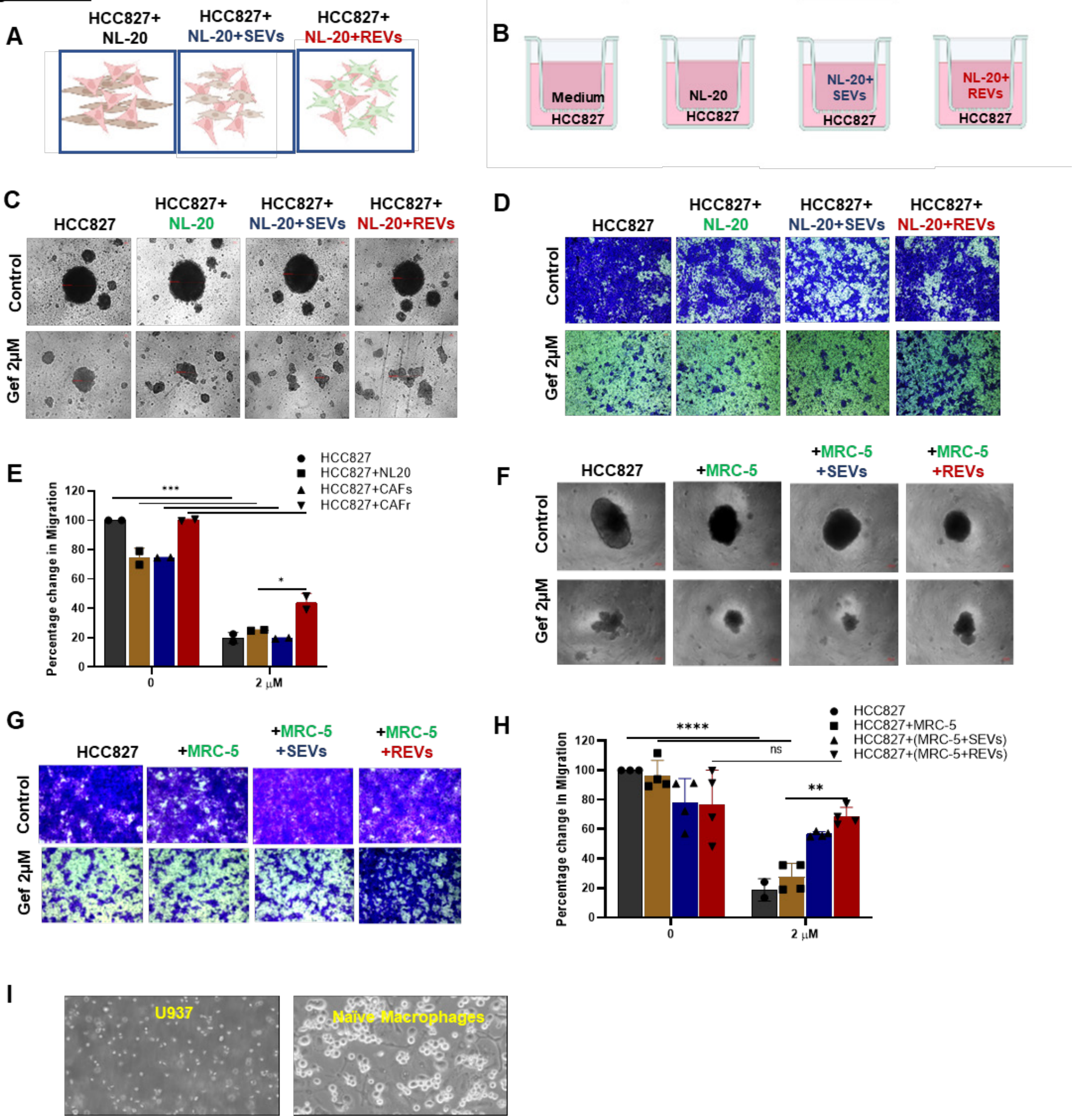

Figure S6: (A, B) Schematic diagram showing co-culture of HCC827 with NL-20 or MRC-5 with and without exposure to SEVs and REVs. **(C, F)** Effect on spheroid formation was assessed in the co-culture (according to B) cells after treatment with gefitinib for 24 hours; 3000 cells were re-seeded on low attachment spheroid plates and left for 7-10 days before viewing under the microscope using 10X magnification (Scale bar: 50 µM). **(D, G)** From the same setup, 75,000 cells were re-seeded in ThinCert® cell culture inserts for 48 hours and stained with crystal violet, and viewed under a microscope (Scale bar: 50 µm) and **(E, H)** quantified by dissolving in 33% (v:v)

acetic acid followed by measuring absorbance at 590 nm as described in Materials and Methods. Migration rates are plotted in percentages with respect to control cells. Data are representative of at least 3 independent experiments and shown as mean ± SD of biological triplicates. Two-way ANOVA was employed for statistical significance (*p < 0.05, **p < 0.01, ***p < 0.001, ****p < 0.0001). **(I)** Naïve macrophages were obtained by treating U937 cells with PMA (10 µg/ml for 48 hours) and phenotypic change was assessed by bright field microscopy (Scale bar: 50 µm).

Figure S7:

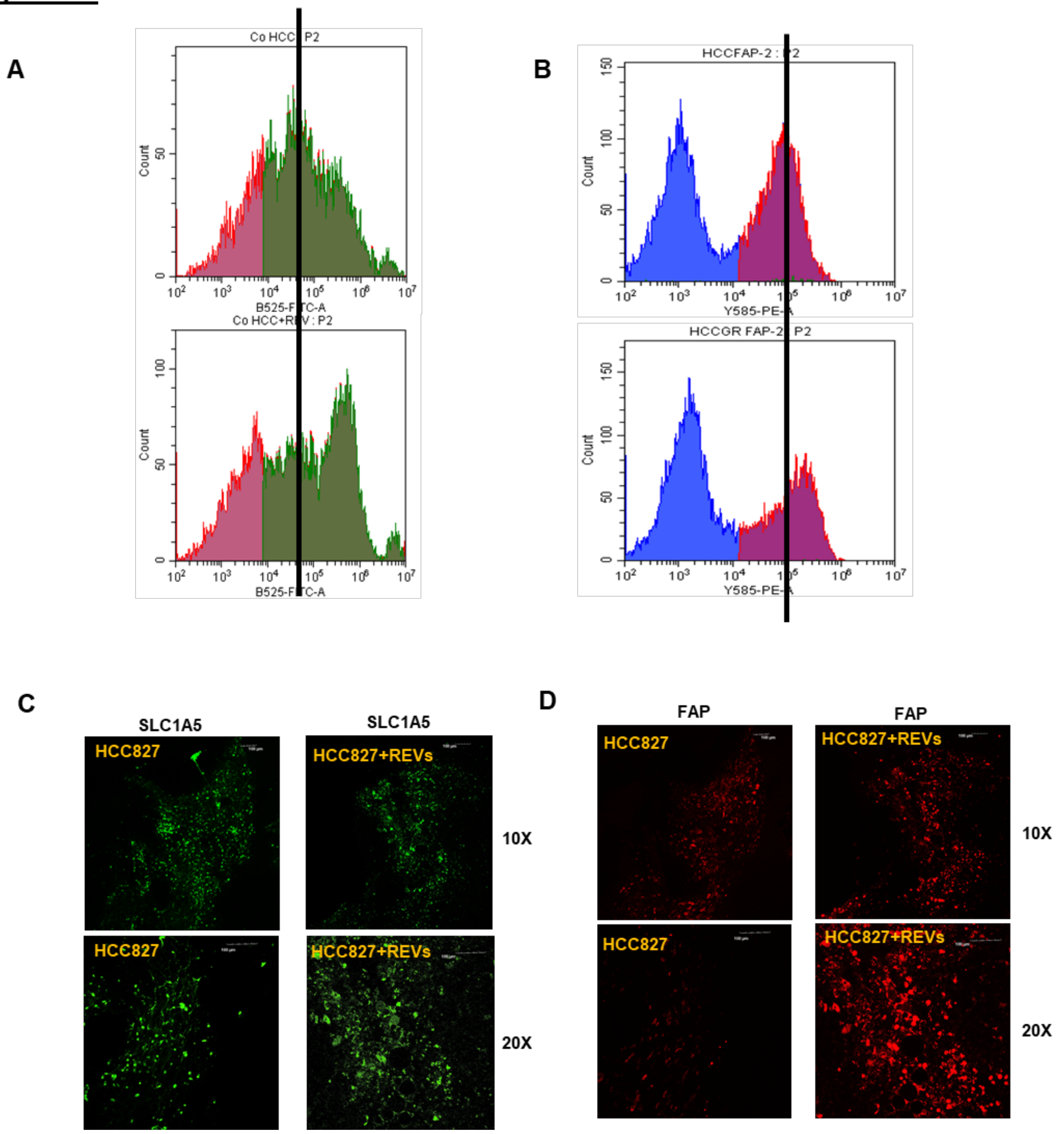

Figure S7: (A) Expression of SLC1A5 and **(B)** FAP on single cell suspension of HCC827 xenograft slices after exposing to REVs for 48 hours was assessed by flow cytometry. At least 10,000 cells were analyzed by flow cytometry as described in Materials and Methods. **(C, D)** Surface expression of SLC1A5, and FAP in HCC827 xenograft slices after exposure to REVs for 48 hours was also assessed by confocal microscopy (Magnification: 10X and 20X; Scale bar: 20 μM).

Supplementary Tables:

Accession	Gene Name	Description	Sum (Coverage)	Sum (# Peptides)	HCC827_GR_Exo (AB)/HCC827_Exo (AB)
Q8WUM4	ALIX	Programmed cell death 6-interacting protein	52.53%	40	1.081
Q99816	TSG101	Tumor susceptibility gene 101 protein	33.59%	12	1.153
B4DPP0	CD9	cDNA FLJ51032, highly similar to CD9 antigen	38.30%	6	1.312
H0YD13	CD44	CD44 antigen	20.87%	4	1.314
F8VV56	CD63	CD63 antigen	9.66%	2	1.123
E9PJK1	CD81	Tetraspanin-28	21.82%	2	0.785

Table S1. Summary of proteins in the EVs of HCC827 cells. EV-related protein details in the EVs isolated from the supernatant of HCC827 cells and identified by proteomics analysis

Accessin	Gene Name	Description	Sum (Coverage)	Sum (Peptide)	TKI resistant NSCLC Plasma /Healthy Plasma Exo	Treated NSCLC Plasma/NSCLC Plasma Exo	TKI Resistant pooled Plasma/Healthy Plasma Exo	Treated pooled Plasma/Pooled Plasma Exo
Q9BRS2	RIOK1	Serine/threonine-protein kinase	45.42%	29	4.613	1.756	4.013	1.304
O75340-2	PDCD6	Programmed cell death protein 6	42.33%	7	1.24	0.902	1.772	1.402
P02786	TFRC	Transferrin receptor protein 1	59.74%	52	2.278	0.628	1.529	0.855
P01031	C5	Complement C5	60.38%	107	1.434	0.771	1.502	0.696
P01024	C3	Complement C3	87.25%	174	1.328	0.811	1.465	0.738
Q12805-3	EFEMP1	EGF-containing fibulin-like extracellular matrix protein 1	40.85%	13	1.432	0.833	1.322	1.124
O00391	QSOX1	Sulfhydryl oxidase 1	22.49%	15	1.354	0.784	1.282	0.895
Q6IC98	GRAMD4	GRAM domain-containing protein 4	25.61%	21	1.301	0.813	1.275	0.839
Q15758	SLC1A5	Neutral amino acid transporter	16.08%	7	2.093	0.664	1.212	0.979
P35908	KRT2	Keratin, type II cytoskeletal 2 epidermal	57.12%	44	1.585	2.633	1.203	0.643
Q8WXI7	MUC16	Mucin-16	25.01%	243	1.814	1.134	1.956	1.28
P12259	F5	Coagulation factor V	38.17%	92	1.239	0.864	1.476	0.873
P13645	KRT10	Keratin, type I cytoskeletal 10	56.85%	36	1.761	2.361	1.266	0.683
B2R950	B2R950	pregnancy-zone protein (PZP)	47.5%	70	1.541	0.599	1.591	0.588
Q6LAM1	Q6LAMI	Heavy chain of factor I	59.81%	28	1.278	0.751	1.284	0.86

Table S2. Summary of commonly upregulated proteins in the EVs of TKI-resistant human plasma: Commonly upregulated proteins in the EVs isolated from the plasma of patients carrying TKI-resistant tumors in comparison with EVs from plasma of healthy volunteers
